# Hybrid fitness effects modify fixation probabilities of introgressed alleles

**DOI:** 10.1093/g3journal/jkac113

**Published:** 2022-05-10

**Authors:** Aaron Pfennig, Joseph Lachance

**Affiliations:** School of Biological Sciences, Georgia Institute of Technology, Atlanta, GA 30332, USA; School of Biological Sciences, Georgia Institute of Technology, Atlanta, GA 30332, USA

**Keywords:** adaptive introgression, Dobzhansky–Muller incompatibilities, heterosis, hybrid vigor, hybrid breakdown, hybrid fitness effects, theoretical population genetics

## Abstract

Hybridization is a common occurrence in natural populations, and introgression is a major source of genetic variation. Despite the evolutionary importance of adaptive introgression, classical population genetics theory does not take into account hybrid fitness effects. Specifically, heterosis (i.e. hybrid vigor) and Dobzhansky–Muller incompatibilities influence the fates of introgressed alleles. Here, we explicitly account for polygenic, unlinked hybrid fitness effects when tracking a rare introgressed marker allele. These hybrid fitness effects quickly decay over time due to repeated backcrossing, enabling a separation-of-timescales approach. Using diffusion and branching process theory in combination with computer simulations, we formalize the intuition behind how hybrid fitness effects affect introgressed alleles. We find that hybrid fitness effects can significantly hinder or boost the fixation probability of introgressed alleles, depending on the relative strength of heterosis and Dobzhansky–Muller incompatibilities effects. We show that the inclusion of a correction factor (*α*, representing the compounded effects of hybrid fitness effects over time) into classic population genetics theory yields accurate fixation probabilities. Despite having a strong impact on the probability of fixation, hybrid fitness effects only subtly change the distribution of fitness effects of introgressed alleles that reach fixation. Although strong Dobzhansky–Muller incompatibility effects may expedite the loss of introgressed alleles, fixation times are largely unchanged by hybrid fitness effects.

## Introduction

Hybridization of related (sub-)species is a common phenomenon in nature, particularly among plants but also animals (Mallet 2005; Abbott *et al.* 2013; Arnold *et al.* 2015; Suvorov *et al.* 2021). For instance, early modern humans interbred with archaic hominins on multiple occasions, e.g. with Neanderthals, leading to ∼2% Neanderthal DNA in contemporary genomes of non-Africans (Green *et al.* 2010; Sankararaman *et al.* 2012; Prüfer *et al.* 2017; Browning *et al.* 2018; Dannemann and Racimo 2018). For this reason, introgression is a crucial source of genetic variation (Hedrick 2013). The evolutionary dynamics of introgressed alleles, however, are different from the dynamics of de novo mutations. This is because introgressed alleles are found in a novel context: hybrid genomes. This novel genomic context gives rise to hybrid fitness effects (HFEs), i.e. interactions between unlinked alleles from different source populations. HFEs influence hybrid fitness, and thereby can affect the evolutionary fates of introgressed alleles independently of direct selection acting on an individual locus (Edmands 2002; Geneva and Garrigan 2010; Kim *et al.* 2017; Dagilis *et al.* 2019; MacPherson *et al.* 2020; Schneemann *et al.* 2020; Moran *et al.* 2021). Thus, accurate models of introgression should account for HFEs.

High hybrid fitness is sometimes observed when two divergent populations interbreed, especially in F1 hybrids (Burke and Arnold 2001; Escobar *et al.* 2008; Wang *et al.* 2015). This hybrid vigor, or heterosis, can be explained by competing but not mutually exclusive theories of dominance, overdominance, and pseudo-overdominance (Lippman and Zamir 2007). According to the frequently invoked dominance theory, introgressed alleles can induce hybrid vigor by masking recessive deleterious alleles in hybrids (Lynch 1991; Birchler *et al.* 2006).

Hybrids can also have low fitness, particularly in the first few generations of back-crossed hybrids (BC1, BC2, etc.)—a phenomenon that is known as hybrid breakdown (Coyne and Orr 2004; Bomblies and Weigel 2007; Edmands 2007; Fang *et al.* 2012; Snoek *et al.* 2014; Vaid and Laitinen 2019). This hybrid breakdown can be induced by introgression introducing segregated variants into a new genomic context, which may cause so-called Dobzhansky-Muller incompatibilities (DMIs) (Kondrashov *et al.* 2002). DMIs arise from untested epistatic interactions of two or more alleles (Dobzhansky 1937; Muller 1942; Fraïsse *et al.* 2014; Presgraves and Meiklejohn 2021), i.e. interactions between alleles from different source populations. Theory predicts that DMIs accumulate faster than linearly with time; thus, DMIs *snowball* (Orr 1995). Due to the accumulation of DMIs, hybridization between highly divergent populations can result in less fit, sterile, or inviable offspring (Orr and Turelli 2001).

Previous theoretical work concerning introgression has mainly focused on linked deleterious alleles that constitute a barrier to introgression (Barton and Bengtsson 1986; Ghosh *et al.* 2012; Uecker *et al.* 2015; Sachdeva and Barton 2018a,b). Models using a branching process framework showed that a beneficial allele that is linked to deleterious alleles can only reach high frequencies if haplotypes are quickly broken up by recombination. Hence, successful introgression largely depends on the dynamics during the early generations after hybridization (Uecker *et al.* 2015). Furthermore, models examining the impact of polygenic selection on introgression under an infinitesimal model with linkage showed that the dynamics of a haplotype block’s introgression depend on its size, genetic variance, and its associated trait value (Sachdeva and Barton 2018a). Some previous theoretical work also dealt with unlinked alleles constituting a barrier to the introgression of a marker allele. Bengtsson formalized the strength of such a genetic barrier—quantified by the so-called “gene flow factor” to the introgression of an unlinked marker allele for different scenarios, including a scenario of a two-locus DMI. The gene flow factor quantifies the reduction in the transmission probability of an introgressed gene due to unlinked genetic incompatibilities (Bengtsson 1974, 1985). However, heterosis and DMI effects often occur at the same time, leading to more complex dynamics (e.g. hybrid vigor followed by hybrid breakdown) (Edmands 1999; Rhode and Cruzan 2005; Stelkens *et al.* 2015). Therefore, accounting simultaneously for polygenic heterosis and DMI effects constitutes an important step forward in the context of how introgressed alleles behave in a new genomic background.

In this paper, we propose a holistic framework that explicitly accounts for unlinked heterosis and DMI effects at any strength in addition to single-locus fitness effects when tracking the fate of a rare introgressed marker allele (*B*). These HFEs are assumed to be highly polygenic, and thus the overall strength of HFEs depends on the fraction of introgressed DNA in a hybrid genome. Repeated backcrossing of hybrids within the recipient population dilutes the amount of introgressed DNA in individual genomes, leading to nonconstant hybrid fitness and a separation of timescales. We formalize the intuition behind how HFEs affect introgressed alleles by providing expressions for the fixation probability using diffusion and branching process theory. Using these approximations together with computer simulations, we address two main questions: first, how do HFEs behave during the first few generations following hybridization? Second, what are the long-term consequences of heterosis and DMI effects concerning the introgression of a single allele?

## Methods

We consider a rare introgression event between donor and recipient populations. Moreover, we suppose that the recipient population has a constant effective population size (*N_e_*), as well as discrete, nonoverlapping generations [i.e. we assume a Wright–Fisher (WF) model]. Individuals are assumed to be diploid. Secondary contact occurs at time *t *=* *0, yielding a single admixed individual at time *t *=* *1, who has one parent from the donor population and one parent from the recipient population. Subsequent generations involve repeated backcrossing of the hybrid(s) within the recipient population. Assuming an infinite number of chromosomes and loci and a large effective population size, this halves the amount of introgressed material in hybrid genomes every generation.

### General model

Our model focuses on a single biallelic autosomal locus. Prior to hybridization, the recipient population is fixed for the *A* allele, and the donor population is fixed for the *B* allele. Mutation rates are assumed to be negligible (i.e. $2N_{e}\mu \ll 1$). We describe the evolutionary dynamics of a semidominant introgressed *B* allele, which depend on locus-specific fitness effects as well as HFEs. We assume that heterosis and DMI effects are polygenic as per empirical results from True *et al.* (1996) and Presgraves and Meiklejohn (2021), implying that each additional unlinked effect makes an infinitesimal contribution to the overall strength of HFEs. Due to repeated backcrossing within the recipient population, introgressed donor DNA is diluted each generation. This dilution induces a decay of the strength of heterosis and DMI effects, resulting in nonconstant hybrid fitness.

The core of our model involves a set of time-dependent fitness functions. In classical population genetics, the fitness of *AA* homozygotes is one, the fitness of *AB* heterozygotes is 1+s, and the fitness of *BB* homozygotes is 1+2s, assuming additive allelic effects (semidominance) at a single locus. As pointed out above, however, we need to account for genome-wide heterosis and DMI effects. For this reason, we introduce the additional parameters *η* and *δ*, describing the strength of heterosis and DMI effects, respectively. Heterosis and DMI effects are antagonistic evolutionary forces and are independent of direct selection acting on the *B* allele. Depending on their relative strength, heterosis and DMI effects can qualitatively alter the selective forces acting on the introgressed *B* allele by modulating the overall hybrid fitness. The time-sensitivity of the fitness functions is expressed by the subscript *t*, which refers to the number of generations following hybridization:

(1) $$w_{AA,t}\approx 1$$

(2) $$w_{AB,t}=(1+s)\times (1+\eta_{t}+\delta_{t})$$

(3) $$w_{BB,t}=(1+2s)\times (1+\eta_{t}+\delta_{t})$$

Here, *η_t_* are the heterosis effects at time *t*, and *δ_t_* is the effect size of the DMI effects at time *t* with t≥1. $w_{AB,t}$ and $w_{BB,t}$ are the fitness of hybrids being heterozygous and homozygous for the introgressed *B* allele, respectively. The fitness of hybrids carrying the *B* allele depends on the intrinsic fitness effects of the *B* allele (1+s and 1+2s) as well as genome-wide HFEs ($1+\eta_{t}+\delta_{t}$). Because we consider a rare introgression event, HFEs are expected to have a minimal effect on the fitness of *AA* individuals ($w_{AA,t}$) in large populations.

Traditionally heterosis effects confer a fitness advantage ($\eta_{t}\geq 0$), while DMI effects have a detrimental effect on fitness ($\delta_{t}<0$), and we will focus on such cases. However, our framework also holds when $\eta_{t}<0$ and $\delta_{t}>0$. The only constraint is that the HFEs term must be greater than or equal to zero ($1+\eta_{t}+\delta_{t}\geq 0$). This is because—paraphrasing Dobzhansky—a fitness less than zero is a “fate worse than death” (Dobzhansky and Pavlovsky 1953). Therefore, when the HFEs term evaluates to a value less than or equal to zero (*η_t_* + $\delta_{t}\leq -1$), HFEs result in sterility and/or lethality, and hybrid fitness is zero. Furthermore, the model is easily extended to other dominance levels of the marker allele by changing the left-hand term in Equations (2) and (3).

Due to the time dependence of heterosis and DMI effects, their decay functions are also critical to our model. Their derivations are briefly delineated below, and the detailed logic leading to the decay functions is explained in the Supplementary Materials.

### Decay of heterosis effects due to backcrossing

**Fig. 1.**
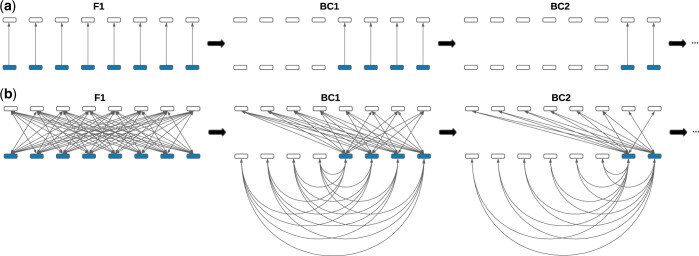
Illustration of the decay of genomewide HFEs. Repeated backcrossing of hybrids within the recipient population (white) halves the amount of introgressed DNA (blue) in hybrid genomes every generation, inducing a decay of HFEs. a) Here, we assume that heterosis effects arise from the masking of recessive deleterious alleles. Heterosis effects halve every generation. b) DMI effects can occur between any possible pair of (semi-)dominant introgressed and semidominant nonintrogressed alleles. Straight lines correspond to possible interactions between unlinked alleles inherited from different parents, while curved lines correspond to interactions of alleles inherited from the same parent. Note that DMI effects decay slower than heterosis effects.

We presume that heterosis is the consequence of genome-wide masking of population-specific recessive deleterious alleles (Fig. 1a) as per the dominance theory (Charlesworth and Willis 2009; Lohr and Haag 2015). Our model assumes that masked deleterious alleles are evenly distributed across the genome and that their effects are additive so that the strength of heterosis effects (*η_t_*) is directly proportional to the amount of introgressed genetic material. Due to repeated backcrossing, the amount of introgressed DNA in hybrid genomes halves every generation. This, in turn, causes heterosis effects to halve every generation. Given an initial strength of heterosis effects in the F1 hybrid (*η*_1_), the strength in BC1 hybrids is $\frac{1}{2}\eta_{1},\;\frac{1}{4}\eta_{1}$ in BC2 hybrids, and so on. Thus, the decay of heterosis effects is given by:

(4) $$\eta_{t}=\eta_{1}\times 2^{-(t-1)}$$

### Decay of DMI effects due to backcrossing

Prior to hybridization, recipient and donor populations independently accumulate substitutions that become fixed. We assume that these substitutions are uniformly distributed along the genome and are not harmful in their respective population. However, when such mutations are introduced into a new genomic context, e.g. by admixture, they can yield untested epistatic interactions, which may cause incompatibilities (Orr 1995). We consider all possible pairwise interactions between unlinked introgressed and nonintrogressed alleles. The number of possible interactions, and thus the strengths of DMI effects decay over time due to the repeated backcrossing (Fig. 1b). Further, we presume that the effects of individual DMIs are multiplicative. As we show in the Supplementary Materials, the multiplicative model is approximated by an additive model if the effect of each DMI is small. This is a valid assumption given the polygenic nature of DMI effects (True *et al.* 1996; Presgraves and Meiklejohn 2021).

In the main text, we consider a model of epistatic interactions between unlinked semidominant introgressed and semidominant nonintrogressed alleles. While the dynamics are identical if introgressed alleles are dominant, no DMI effects are observed if introgressed alleles are recessive because the chance of hybrids being homozygous for introgressed alleles is negligible in the scenario of a rare introgression event. However, the dynamics differ for other dominance levels of nonintrogressed alleles. We limit the discussion of such epistasis models to the Supplementary Materials, as the qualitative effects are similar.

In hybrids, any pair of introgressed (blue) and nonintrogressed (white) alleles that are not allelic copies can epistatically interact. As we assume a rare introgression event, most such interactions will take place between introgressed and nonintrogressed alleles inherited from different parents (straight lines in Fig. 1b). However, due to recombination, hybrid parents inherit a mix of introgressed and nonintrogressed alleles, rendering possible interactions between introgressed and nonintrogressed alleles inherited from the same parent (curved lines in Fig. 1b). Therefore, the decay of DMI effects is defined by:

(5) $$\begin{array}{c} \delta_{t}=\delta_{1}\times \left[2^{-(t-1)}+\left(1-2^{-(t-1)}\right)\times 2^{-(t-1)}\right]\\ =\delta_{1}\times \left[(2^{t}-1)2^{2-2t}\right] \end{array}$$

Considering the nonreduced form of Equation (5), the first term in the parenthesis corresponds to the decay of the number of possible interactions between unlinked introgressed and nonintrogressed alleles inherited from different parents. The number of such interactions is halved every generation, as the amount of introgressed genetic material is halved [this is the same as the decay of heterosis effects defined in Equation (4)]. As discussed above, with increasing dilution of the introgressed genetic material, interactions between unlinked introgressed and nonintrogressed alleles inherited from the same parent become possible, which are captured by the second term in the parenthesis. Provided an initial strength of DMI effects in the F1 hybrid (*δ*_1_), the strength is $\frac{3}{4}\delta_{1}$ in BC1 hybrids, $\frac{7}{16}\delta_{1}$ in BC2 hybrids, $\frac{15}{64}\delta_{1}$ in BC3 hybrids, and so forth (see Supplementary Materials for more details). Thus, DMI effects do not halve every generation but decay slightly slower.

### Fixation probability

Here, we take up approximations of the fixation probability using diffusion and branching process theory, respectively.

First, we introduce a correction factor *α*, representing the compounded effect size of HFEs. *α* is similar to the “gene flow factor,” measuring the strength of the genetic barrier against gene flow that arises from unlinked genetic incompatibilities (Bengtsson 1974, 1985). The compounded effect size of HFEs is given by the product of the HFEs term in Equations (1)–(3) over all generations:

(6) $$\alpha =\prod_{t=1}^{\infty }\left(1+\eta_{t}+\delta_{t}\right)$$

**Fig. 2.**
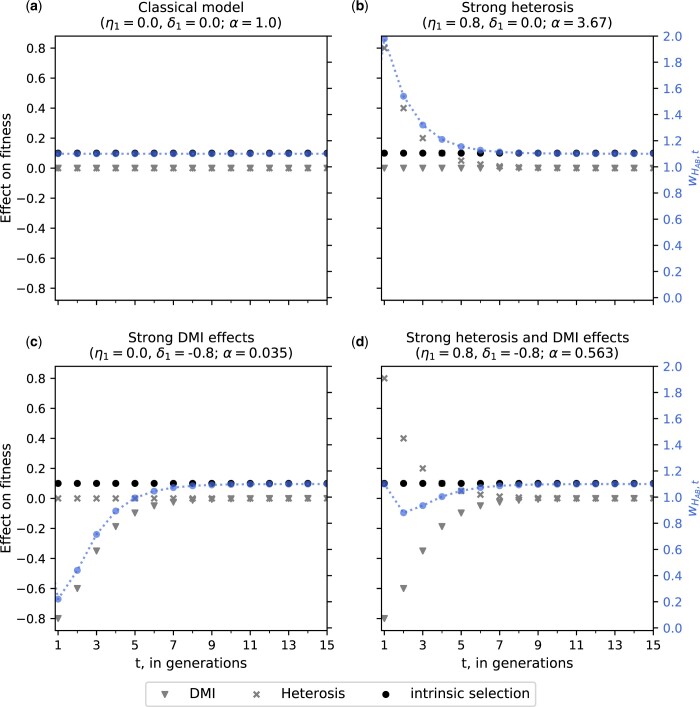
Temporal dynamics of different hybrid fitness scenarios: a) classical model; b) strong heterosis effects; c) strong DMI effects; and d) strong heterosis and DMI effects. HFEs (gray scale) quickly decay and asymptotically approach zero. Therefore, the overall fitness of heterozygotes that carry the introgressed *B* allele (blue) is time-sensitive. The different decay rates of heterosis and DMI effects can capture complex natural phenomena, such as F1-vigor followed by F2-breakdown. For all panels, the intrinsic selection coefficient of the *B* allele (black) is 0.1. *α* is the compounded effect size of HFEs [Equation (6)].

In practice, it is sufficient to compute this product over the first 20 generations, as HFEs asymptotically approach zero (Fig. 2).

Standard population genetic theory predicts that a neutral allele fixes with a probability equivalent to its frequency. For example, a neutral de novo mutation fixes with probability $1/2N_{e}$. A general pattern that arises is that HFEs linearly scale the fixation probabilities of neutral alleles by a factor of *α*, suggesting that HFEs rescale the effective frequency with that a neutral allele enters a population. For instance, a neutral *B* allele that is introgressed as a single copy fixes with probability $\alpha /2N_{e}$ (root mean square error < $10^{-10}$; Supplementary Fig. 1).

Applying similar logic, we re-scale the initial allele frequency in Kimura’s formula for the fixation probability with *α* (Kimura 1962), yielding

(7) $$u(x)=\frac{1-e^{-4N_{e}s\alpha x}}{1-e^{-4N_{e}s}}$$

As we shall see, thereby, we effectively account for HFEs and obtain accurate estimates of the fixation probability when HFEs matter. 

For weakly beneficial mutations and in the limit of a large effective population size (i.e. $N_{e}s<1$), Equation (7) suggests that the fixation probability of a rare introgressed allele is approximately 2αs, reinforcing the idea that HFEs linearly scale the fixation probability.

Fixation probabilities of beneficial alleles can also be obtained from branching processes. Using a time-homogeneous branching process, Haldane (1927) showed that the fixation probability of a weakly beneficial allele is ∼2*s* if it is initially present as a single copy in a large population and the number of offspring alleles is Poisson distributed. However, this standard population genetic theory does not account for time-heterogeneous fitness, e.g. due to HFEs. Because of the time-dependent hybrid fitness, the branching process becomes time-heterogeneous. In the following, we also assume a Poisson distributed number of offspring alleles with an introgressed *B* allele contributing, on average, $w_{AB,t}$ [Equation (2)] offspring alleles in generation *t*. Then, the survival probability (*u_t_*) of the beneficial *B* allele by generation *t* is given by the following time-heterogeneous branching process:

(8) $$\begin{array}{c} u_{t}=1-G_{t}(0)\\ =1-\text{exp}\;\left[\lambda_{0}\left(\ldots \lambda_{t-3}\left(\text{exp}\;\left(\lambda_{t-2}\left(\text{exp}\;\left[-\lambda_{t-1}\right]-1\right)\right)-1\right)\ldots \right)\right] \end{array}$$

where $G_{t}(0)$ is the corresponding extinction probability by generation *t*, and *λ_t_* is the hybrid fitness $w_{AB,t}$ in generation *t*. Equation (8) was derived by Ohta and Kojima (1968) for the survival probability of an inversion with time-varying fitness (see Supplementary Materials for a detailed derivation). Due to the time-varying fitness, the fixation probably must usually be approximated numerically from Equation (8) (Ohta and Kojima 1968; Uecker and Hermisson 2011).

### Computer simulations

We complement our analyses with WF simulations. This involves tracking the frequency of the introgressed *B* allele in a population with constant effective population size (*N_e_*), assuming an initial allele frequency of $1/2N_{e}$. The fitness of the *B* allele in generation *t* is given by Equations (1)–(3). The simulations results are then averaged over many runs.

For results in the main text, we presume an infinite number of chromosomes and loci. A description of simulations where we relax this assumption, i.e. there is stochastic dilution of introgressed DNA, is given in the Supplementary Materials. We also suppose a large *N_e_* (i.e. 10,000) so that the chances of two hybrids mating are negligible during the first few generations after admixture. For these reasons, the amount of introgressed DNA in a hybrid genome halves every generation, and heterosis and DMI effects decay deterministically according to Equations (4) and (5), respectively.

## Results

The evolutionary dynamics of introgressed alleles are affected by polygenic HFEs. Here, we focus on heterosis effects and DMI effects that are due to epistatic interaction between (semi-)dominant introgressed and semidominant nonintrogressed alleles. The dynamics of other epistatic models are explored in the Supplementary Materials.

### Hybrid fitness

We start by analyzing the effect of HFEs on the overall hybrid fitness using Equations (1)–(3) in four exemplary scenarios: (1) absence of HFEs (the classical model); (2) strong heterosis effects; (3) strong DMI effects; and (4) strong heterosis and DMI effects. Generally, HFEs are only relevant during the first few generations after hybridization (1≤t≤20) as both heterosis and DMI effects asymptotically approach zero (compare panels in Fig. 2). After multiple generations of backcrossing, direct selection acting on the introgressed *B* allele becomes the strongest evolutionary force. Because of this fast decay of HFEs, the decay of heterosis effects due to the purging of recessive deleterious alleles from the gene pool by selection can be neglected.

In the absence of HFEs ($\eta_{1}=0$; $\delta_{1}=0$), hybrid fitness is solely determined by direct selection acting on the *B* allele, and our model reduces to the classical WF model (Fig. 2a). When heterosis effects are initially much stronger than DMI effects ($\eta_{1}\gg |\delta_{1}|$), hybrid vigor is observed (Fig. 2b). Similarly, when DMI effects are initially much stronger than heterosis effects ($|\delta_{1}|\gg \eta_{1}$), hybrid breakdown is observed (Fig. 2c). A special scenario is when heterosis and DMI effects have the same initial strength ($\eta_{1}=|\delta_{1}|$). HFEs are absent in the first generation since heterosis and DMI effects balance each other. Due to the faster decay of heterosis effects, however, DMI effects determine hybrid fitness in the following generations, leading to hybrid breakdown (Fig. 2d). Because of the faster decay of heterosis effects, hybrid vigor followed by hybrid breakdown can be observed when heterosis effects are initially moderately stronger than DMI effects, e.g. $\eta_{1}=0.5$ and $\delta_{1}=-0.45$.

### Probability of fixation and compounded HFEs

**Fig. 3.**
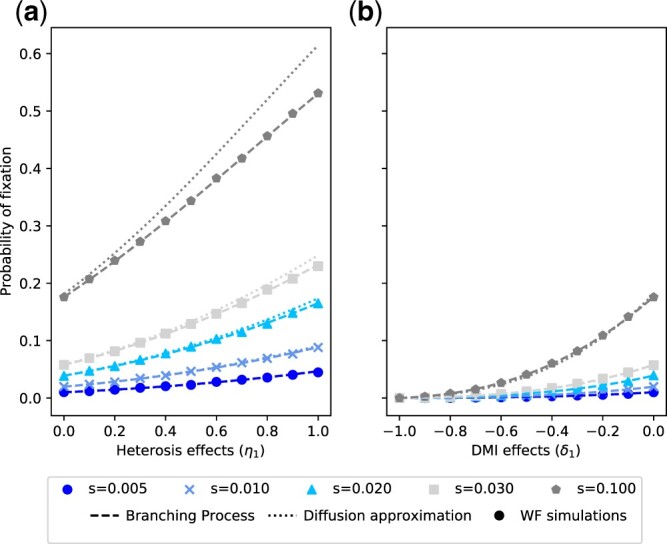
Comparison of the fixation probabilities of the introgressed *B* allele obtained from diffusion approximations [Equation (7)], branching processes [Equation (8)], and WF simulations. a) Only heterosis effects ($\delta_{1}=0.0$) and b) only DMI effects ($\eta_{1}=0.0$). Theoretical fixation probabilities match simulations, with the exception of the diffusion approximation of the fixation probability for strongly selected alleles in the presence of strong heterosis. Fixation probabilities from simulations were computed based on 100,000 runs with $N_{e}=10,000$ and $q_{0}=1/2N_{e}$.

Next, we evaluated how well fixation probabilities of the introgressed *B* allele are approximated by the diffusion approximation [Equation (7)] and the branching process [Equation (8)] by comparing them to fixation probabilities derived from WF simulations. As shown in Fig. 3, both theoretical approaches accurately describe the fixation probability under our model. While fixation probabilities estimated from the branching process match simulations, the diffusion approximation slightly overestimates fixation probabilities if heterosis effects are strong and selection coefficients large (Fig. 3a). However, this scenario is likely to be rare, as we expect most hybrids to suffer from hybrid breakdown and most selection coefficients to be small (Edmands 2007).

Since HFEs linearly scale the fixation probability with their compounded effect size (*α*), the fixation probability is increased when *α* is > 1 and decreased when α is < 1. For weak initial heterosis and DMI effects, Equation (6) can be approximated with $1+\sum_{t=1}^{\infty }\eta_{t}+\sum_{t=1}^{\infty }\delta_{t}$. Then, *α* is approximated by $1+2\eta_{1}+\frac{8}{3}\delta_{1}$, and *α* is ≥1 if heterosis effects are initially at least $\frac{4}{3}$ times as strong as DMI effects. Thus, the fixation probability is increased when $\frac{4}{3}\eta_{1}\geq |\delta_{1}|$. This observation is explained by the different decay rates of heterosis effects and DMI effects. As discussed above, when initial heterosis effects are moderately stronger than DMI effects, hybrid vigor followed by hybrid breakdown is observed, diminishing the fixation probability.

**Fig. 4.**
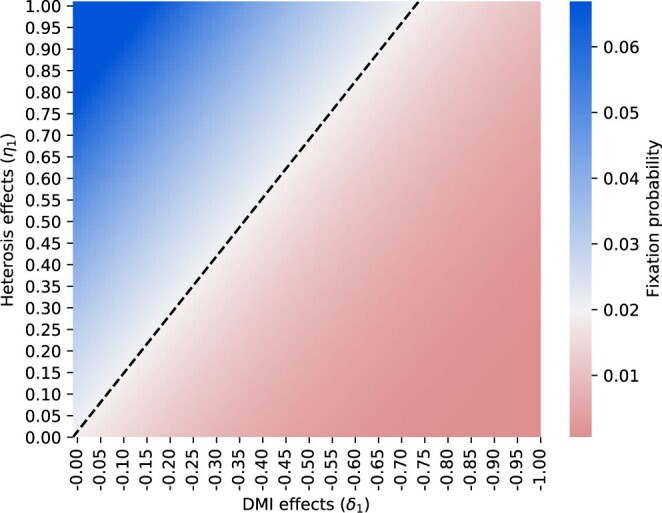
Fixation probabilities for different initial strengths of heterosis and DMI effects. Heterosis effects can increase the fixation probability (top-left corner), while DMI effects can prohibit fixation (bottom-right corner). The black line corresponds to parameter combinations that match fixation probabilities expected from standard population genetics theory (∼2s). Fixation probabilities for the introgressed *B* allele were computed using Equation (8), assuming a selection coefficient *s *=* *0.01.

Further analyses investigated how different parameter values affect the fate of the introgressed *B* alleles. Figure 4 shows the probability of fixation for various combinations of initial strengths of heterosis effects (*η*_1_) and DMI effects (*δ*_1_). Combinations of initial strengths of heterosis effects (*η*_1_) and DMI effects (*δ*_1_) on the dashed black line in Fig. 4 match fixation probabilities expected from standard population genetics theory (i.e. ∼2s). This line divides the parameter space such that parameter combinations above it lead to increased fixation probabilities compared with the classical model (blue colors in the top left corner), while combinations below this line lead to a lower chance of fixation (red colors in the bottom right corner). A linear regression analysis—using absolute values—showed that the slope of this black line is 1.35 ($R^{2}=0.99,\;P<10^{-10}$) if *s* is 0.01. This finding is in agreement with the above approximation for weak HFEs (i.e. $\frac{4}{3}\eta_{1}\geq |\delta_{1}|$).

### HFEs subtly change the distribution of fitness effects of introgressed alleles

What is the distribution of selection coefficients conditioned on fixation? This depends on two factors: the original distribution of fitness effects (DFE) of alleles, which we denote by *f*(*s*), and the corresponding fixation probabilities.

**Fig. 5.**
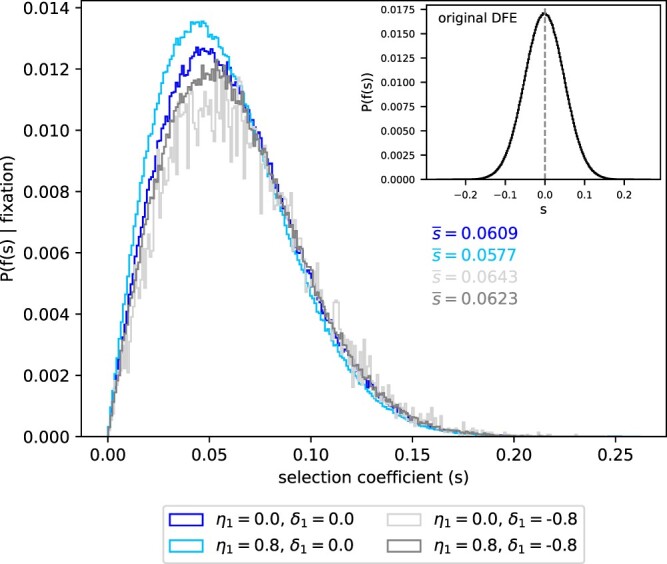
The distribution of fitness effects of introgressed alleles that reach fixation. Heterosis effects subtly shift the DFE toward smaller selection coefficients, while DMI effects shift it toward slightly greater values. The DFEs of fixed alleles was inferred from 10^7^ WF simulations with selection coefficients drawn from the original DFE, which was modeled using a normal distribution with a mean of –0.001 and an SD of 0.05 (see inset). WF simulation parameters: $N_{e}=10,000$ and $q_{0}=1/2N_{e}$.

Previous studies estimated the DFE of deleterious alleles (Eyre-Walker *et al.* 2006; Eyre-Walker and Keightley 2007; Kim *et al.* 2017) and advantageous alleles (Bataillon *et al.* 2011; McDonald *et al.* 2011; Frenkel *et al.* 2014). For the sake of simplicity and illustrative purposes, we chose to describe *f*(*s*) with a normal distribution with a mean of –0.001 and an SD of 0.05. We drew 10,000,000 selection coefficients from this distribution and kept only those that reached fixation in WF simulations. Figure 5 shows the distributions of selection coefficients conditioned on fixation for four different scenarios of HFEs. Note that Fig. 5 does not show the absolute probabilities of introgression. The probabilities of successful introgression are, in fact, reduced when DMI effects are strong (Supplementary Fig. 2).

Heterosis effects increase the fraction of less advantageous alleles that are introgressed, while DMI effects reduce their share among introgressed alleles (Fig. 5). Under the classical model ($\eta_{1}=\delta_{1}=0.0$), the mean value of *s*, conditioned on fixation, is $\bar{s}=0.0609$. Strong heterosis effects ($\eta_{1}=0.8$ and $\delta_{1}=0.0$) shift the DFE of introgressed alleles toward lower values of *s*, i.e. $\bar{s}$ decreases to 0.0578. Given strong DMI effects ($\eta_{1}=0.0$; $\delta_{1}=-0.8$), $\bar{s}$ increases to 0.0634. When heterosis and DMI effects are of similar strength ($\eta_{1}=0.8$; $\delta_{1}=-0.8$), $\bar{s}$ is shifted to 0.0622, which is greater than under the classical model. Similar patterns arise when the original DFE (*f*(*s*)) is exponentially distributed (Supplementary Fig. 3).

### Sojourn times

**Fig. 6.**
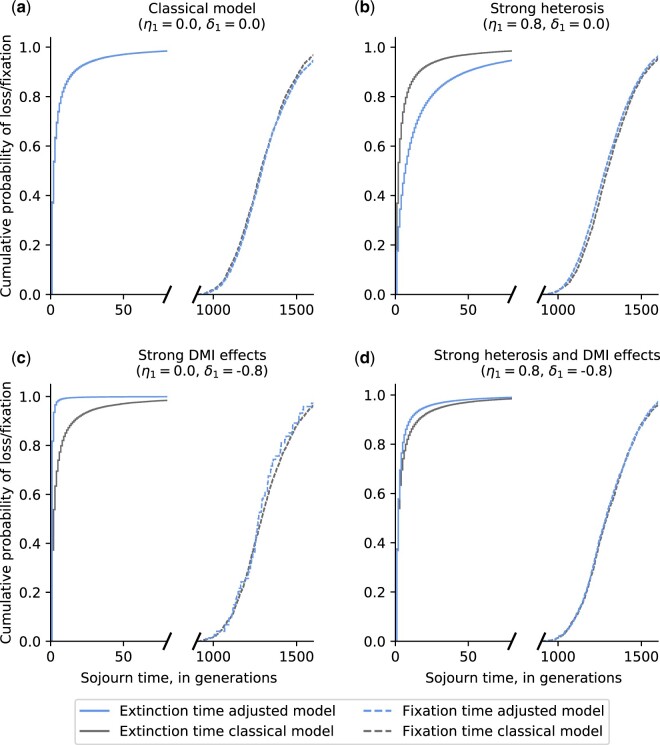
HFEs affect extinction times but not fixation times: a) classical model; b) strong heterosis effects; c) strong DMI effects; and d) strong heterosis and DMI effects. Distributions of sojourn times were inferred based on 100,000 WF simulations with $N_{e}=10,000$, *s *=* *0.01, and $q_{0}=1/2N_{e}$.

We also examined whether HFEs change the timescale of when the introgressed *B* allele becomes lost or fixed, respectively. Sojourn times were compared under our model to the classical model for the four different scenarios previously discussed (Fig. 6).

In general, HFEs have a larger effect on extinction times than fixation times. Heterosis effects have the power to delay the extinction of an allele (Fig. 6b), while DMI effects can accelerate the loss of alleles (Fig. 6c). DMI effects with a strength of –1.0 ($\delta_{1}=-1.0$) are lethal in the absence of heterosis, leading to the immediate extinction of an allele. Consistent with our previous findings concerning hybrid fitness and fixation probabilities, the loss of an allele is marginally expedited when heterosis and DMI effects are of similar strength (e.g. $\eta_{1}=|\delta_{1}|=0.8$; Fig. 6d). Despite heterosis and DMI effects’ power to change the probability of fixation of an introgressed allele, they have negligible effects on the timescale of when an allele reaches fixation.

## Discussion

In this paper, we demonstrated the importance of HFEs when studying admixture. Despite their quick decay due to the dilution of introgressed genetic material by repeated backcrossing, we find that heterosis and DMI effects influence the fixation probabilities of an unlinked introgressed marker allele. The fast decay of HFEs due to repeated backcrossing facilitates a separation-of-timescales approach. Furthermore, our model captures complex phenomena observed in nature, such as hybrid vigor followed by hybrid breakdown, which is explained with heterosis obscuring hybrid breakdown in the early generations following admixture (Edmands 1999; Rhode and Cruzan 2005; Stelkens *et al.* 2015). For instance, if heterosis effects are initially slightly stronger than DMI effects, heterosis effects obscure hybrid breakdown in the F1-generation, leading to F1-vigor and subsequent hybrid breakdown due to the slower decay of DMI effects.

We provided expressions for the fixation probability for rare introgressed alleles by taking into account the compounded effect size of HFEs [*α*, Equation (6)], using diffusion and branching process theory [Equations (7) and (8)]. The diffusion approximation slightly overestimates the fixation probabilities of strongly selected alleles (e.g. *s *=* *0.1) when heterosis effects are strong because the separation of timescales is not as strong but is the more general solution. This is because the diffusion approximation relaxes the assumption of an infinitely large population size, and it also allows estimating fixation probabilities of deleterious alleles. Overall, fixation probabilities depend on the initial strength of heterosis and DMI effects. As the compounded effect size of HFEs (*α*) linearly scales the fixation probability, *α* indicates whether the fixation probability of an introgressed allele is increased or decreased by HFEs, i.e. if *α* > 1 or α < 1, respectively. Furthermore, HFEs affect the fixation probabilities of alleles with small selection coefficients more than of alleles with large selection coefficients due to the weaker intrinsic selection of those alleles.

Given HFEs’ power to change fixation probabilities of introgressed alleles, their impact on the distribution of fitness effects is unexpectedly subtle. The DFE of alleles that reach fixation is only subtly shifted toward smaller selection coefficients by heterosis effects (Fig. 5). Conversely, DMI effects increase the probability density in the right tail of the DFE. Due to the separation of time scales, the shift is only subtle because HFEs (*α*) linearly transform the joint distribution of the original DFE (*f*(*s*)) and fixation probabilities (i.e. 2αsf(s); Supplementary Fig. 2). For the same reasons, the effect of HFEs on the DFE of alleles that reach fixation is independent of the form and shape of the original DFE and holds for both deleterious and advantageous mutations.

We also found that HFEs influence extinction times more than fixation times. This is because the loss of an allele occurs on a short timescale that is similar to the timescale of when HFEs act, whereas fixation occurs on a much longer timescale. HFEs, however, can boost an allele to a frequency at which it is less likely to be lost due to genetic drift. If an allele survives the first 20 generations, our model reduces to the classical model, leaving fixation times largely unaffected. Therefore, HFEs only delay or expedite the loss of an allele but have only slight effects on the time until fixation.

To understand how HFEs delay or expedite the loss of an allele, it is helpful to compare their action to those of enzymes. Enzymes catalyze a reaction by reducing the activation energy—the hurdle—to get a reaction started. Once the reaction is started, it carries out by itself. Similarly, heterosis effects reduce the hurdle for an allele to become established by reducing the relative strength of genetic drift. Once an allele is established, its intrinsic selection takes over, eventually driving it toward fixation. Contrarily, DMI effects increase the hurdle, rendering it harder for an allele to become established; thereby, increasing the likelihood of an allele’s loss.

If the assumption of an infinite number of chromosomes and loci is violated, the amount of introgressed DNA in individual hybrid genomes does not exactly halve every generation. Although the differences in fixation probability in simulations with deterministic and stochastic dilution of introgressed DNA (see Supplementary Materials) are statistical significant in a few cases, the absolute fixation probabilities are only marginally different between the deterministic and stochastic dilution model of introgressed DNA (Supplementary Table 1). Thus, the conclusions presented in this paper are robust to violations of the assumption of an infinite number of chromosomes and loci.

We only considered the introgression of a single copy of the *B* allele into a large population throughout this paper. This allowed a clear assessment of the impact of HFEs and guaranteed comparability to standard population genetics theory. For bigger introgression pulses, the dynamics are more complex as the amount of introgressed DNA does not necessarily halve every generation, slowing the decay of HFEs. This is because the interbreeding of two hybrids is likelier during the first few generations after admixture. The slower decay would inflate the overall effects of HFEs. Introgressed DNA may also be under selection itself and affect the fitness of *AA* individuals, further complicating the dynamics. Thus, bigger introgression pulses would require tracking introgressed DNA in individual genomes.

Furthermore, the dynamics of our model depend on the initial strength of heterosis and DMI effects. However, it is difficult to obtain accurate estimates of these parameters because HFEs are influenced by genome composition, environmental circumstances, and the history of the admixing populations (Edmands 2002; Geneva and Garrigan 2010; Dagilis *et al.* 2019; MacPherson *et al.* 2020; Schneemann *et al.* 2020; Brice *et al.* 2021; Moran *et al.* 2021). For these reasons, more theoretical and empirical work is needed to better predict the impact of HFEs in cases of admixture.

In many cases of admixture we expect initial DMI effects to be stronger than heterosis effects, given that DMIs accumulate with the square of time (Orr 1995; Edmands 2007; Fang *et al.* 2012). Thus, our results suggest that HFEs can constitute an obstacle toward introgression, as introgressed alleles must survive a DMI filter. Projecting this to the interbreeding of modern humans with Neanderthals, our results suggest that introgressed archaic alleles survived this DMI filter. This DMI filter and the high mutational load in Neanderthals (Harris and Nielsen 2016; Steinrücken *et al.* 2018) hint that introgressed alleles in contemporary human genomes are presumably not that harmful.

By simultaneously accounting for antagonistic heterosis and DMI effects and explicitly modeling their decay, the work presented here expands on previous theory that dealt with the strength of a genetic barrier to gene flow that arises from unlinked genetic incompatibilities (Bengtsson 1974, 1985; Barton and Bengtsson 1986). Our work is also complementary to previous theoretical work, which focused on the effects of linked deleterious alleles and polygenic selection on introgression (Uecker *et al.* 2015; Sachdeva and Barton 2018a). Therefore, models simultaneously accounting for polygenic HFEs arising from interactions between unlinked loci and effects of linkage constitute interesting directions for future work.

Altogether, our results state the importance of considering HFEs when studying introgression. Because HFEs are compounded, initially moderate hybrid fitness effects—especially DMIs—can have a large impact on the fate of introgressed alleles. More generally, our results emphasize the importance of accounting for the genomic context in which alleles occur when calculating fixation probabilities.

## Data availability

Python3 code used for the simulations and generating the figures can be found at https://github.com/LachanceLab/introgression_theory. The authors state that all data necessary for confirming the conclusions presented in the article are represented fully within the code.

Supplemental material is available at *G3* online.

## Supplementary Material

jkac113_Supplementary_Data
