# Supplementary material for: Hybrid fitness effects modify fixation probabilities of introgressed alleles
Source: G3 (Bethesda). 2022 May 10;12(7):jkac113. doi: 10.1093/g3journal/jkac113 (PMC9258535; doi:10.1093/g3journal/jkac113)
Supplement: jkac113_Supplementary_Data [file jkac113_supplementary_data.pdf]

Supplementary Materials

Hybrid fitness effects modify fixation probabilities of introgressed alleles

Aaron Pfennig[1] and Joseph Lachance[1]

[1]School of Biological Sciences, Georgia Institute of Technology, Atlanta, GA 30332, USA

The decay of hybrid fitness effects explained

We assume that hybrid fitness effects (HFEs) arise from genome-wide interactions between unlinked introgressed and non-introgressed alleles that are independent of direct selection acting on a single locus. We suppose diploid genomes with an infinite number of chromosomes and loci, i.e., an infinitesimal model. Further, we assume that, upon hybridization, one set of chromosomes with n loci introgresses from the donor population into the recipient population, i.e., the mating of one individual from the recipient and donor population. The recipient population is assumed to have a large effective population size so that the probability of two hybrids mating is virtually zero during the early generations following hybridization. Therefore, subsequently repeated backcrossing of the hybrid(s) within the recipient population halves deterministically the fraction of initially introgressed DNA in hybrid genomes every generation (F1 $\rightarrow$ 100%, BC1 $\rightarrow$ 50%, BC2 $\rightarrow$ 25%, etc.). If the number of loci n is finite, the amount of introgressed DNA in hybrids does not always halve every generation; hence, the decay of HFEs is subject to stochasticity. Although the differences in fixation probability in simulations with deterministic and stochastic dilution of introgressed DNA are statistically significant in a few cases, the fixation probabilities are only marginally different between the deterministic and stochastic dilution model of introgressed DNA (Table S1; see Stochastic dilution of introgressed DNA below). Thus, the dynamics delineated below hold even if the assumption of an infinite number of chromosomes and loci is violated. For larger introgression pulses, the dynamics are more complicated because the dilution of introgressed DNA in hybrids occurs slower, inflating the overall effects of HFEs.

DMI effects are assumed to arise from pairwise interactions between unlinked introgressed and non-introgressed alleles. We consider all possible pairwise interactions between the n introgressed loci and the n non-introgressed loci but exclude interactions between allelic copies. If introgressed alleles are semidominant or dominant and non-introgressed alleles are semidominant, this amounts to $n \times (n-1)$ possible interactions in the F1 hybrid (Figure 1B). As we assume the infinitesimal model, the number of possible interactions in the F1 hybrid is approximately n^2. Inevitably, all these interactions are between alleles inherited from different parents. For a discussion of other dominance levels of non-introgressed alleles, see Different epistasis models leading to DMI effects below.

As the amount of introgressed DNA halves every generation, the number of possible interactions between unlinked introgressed and non-introgressed alleles that are inherited from different parents halves. However, because of recombination, hybrid parents inherit introgressed and non-introgressed alleles, between which it can also come to interactions. $\frac{1}{2}n$ introgressed loci can interact with n non-introgressed loci inherited from different parents (straight lines in Figure 1B) and with $\frac{1}{2}n$ non-introgressed loci inherited from the same parent (curved lines in Figure 1B) in BC1 hybrids, summing to $(n \times \frac{1}{2}n) + (\frac{1}{2}n \times \frac{1}{2}n) = \frac{3}{4}n^2$ possible interactions. Extending this logic to BC2 and BC3 hybrids, we obtain $\left(\frac{1}{4}n \times n\right) + \left(\frac{3}{4}n \times \frac{1}{4}n\right) = \frac{7}{16}n^2$ and $\left(\frac{1}{8}n \times n\right) + \left(\frac{7}{8}n \times \frac{1}{8}n\right) = \frac{15}{64}n^2$ possible interactions, respectively.

If we now assume an equal probability for all possible interactions to form a DMI (p_I), an equal absolute effect size c for all DMIs, and a multiplicative model, the strength of DMI effects decays with the number of possible interactions. The strength of DMI effects at time t is given by:

$$\delta_t = (1-c)^{n^2 p_I g_t} - 1 \tag{S1}$$

where g_t is the fraction of possible interactions that are possible in hybrid genomes t generations after admixture, and the -1 term ensures that $\delta_t \leq 0$.

Representing the expected number of DMIs $n^2 p_I$ in the F1 hybrid with m and making use of the Taylor series, Equation S1 becomes

$$\delta_t = -mcg_t + \frac{mc^2 g_t \times (mg_t - 1)}{2} - \frac{c^3 \times (mg_t \times (mg_t - 2) \times (mg_t - 1))}{6} + O(c^4) \tag{S2}$$

for small c. As DMI effects are expected to be polygenic (True *et al.* 1996; Presgraves and Meiklejohn 2021), it is valid to assume that the effect size of an individual DMI (c) is small. Therefore, terms of $O(c^2)$ and greater can be ignored, and Equation S2 is approximated by $-mcg_t$. This is equivalent to an additive model with initial strength $(1-c)^m - 1 \approx -mc$. Then, Equation 5 in the main text is obtained by representing the initial strength of DMI effects $-mc$ with δ_1 and substituting g_t with the decay rate of possible interactions.

Heterosis effects are assumed to arise from the masking of recessive deleterious alleles, i.e., the dominance theory. The same reasoning as above for DMI effects applies to the decay of heterosis effects. The number of possibly masked recessive alleles is directly proportional to the amount of introgressed DNA in hybrid genomes, i.e., n, $n/2$, $n/4$, and $n/8$ in F1, BC1, BC2, and BC3 hybrids, respectively. Assuming an equal probability for each introgressed allele to mask a recessive deleterious allele (p_M), an equal effect size z for each masking event, and an additive model, we can calculate the strength of heterosis effects at time t (η_t) with respect to their initial strength ($np_M z = \eta_1$) (Equation 4 in the main text).

Stochastic dilution of introgressed DNA

To simulate the stochastic dilution of introgressed DNA, we explicitly track the fraction of introgressed DNA present in an individual at a given time t relative to the amount of introgressed DNA present in the F1 hybrid.

For reproduction, two parents are randomly picked with a probability equivalent to their fitness. To avoid selfing, it is ensured that the parents are different individuals. The total amount of introgressed DNA in the child is then the sum of the amount of introgressed DNA it inherited from each parent. Assuming a genome size of 3.2 Gb, the genome can be divided into 32 chunks of 100 Mb, given that one centimorgan is approximately 1 Mb. For each chunk, a diploid individual has two haplotypes - one from each of their parents - and the probability that a parent passes on a specific haplotype to their child is 0.5. For this reason, the probability that a specific set of haplotypes that trace to the same grandparent is inherited to the next generation is modeled with a binomial distribution with 32 trials and a probability of success of 0.5 (the effects of different genome sizes can be explored using the code available at https://github.com/LachanceLab/introgression_theory). Given the parents relative amount of introgressed DNA is $Y_{p,i}$, the probability distribution of the relative amount of introgressed DNA in the offspring (Y_o) is then

$$Y_o = \sum_{i=1}^{2} \frac{Y_{p,i} \times \binom{32}{N} 0.5^N (1-0.5)^{32-N}}{32} \quad \text{(S3)}$$

The strength of heterosis and DMI effects in a given offspring at time t is then given by $\eta_t = \eta_1 \times Y_o$ and $\delta_t = \delta_1 \times (Y_o + Y_o \times (1 - Y_o))$, respectively. In each generation, the entire population is replaced by their offspring, i.e., the Wright-Fisher model.

Given that we consider a rare introgression event, that is, the hybridization of a single individual from the recipient and donor population, the chances of individuals with introgressed DNA mating are negligible in large populations during the first few generations after admixture; hence, the effects on the decay of HFEs are negligible, too. However, in small populations, it is more likely that two individuals with introgressed DNA are mating during the first few generations, slowing the dilution of introgressed DNA, and hence also slowing the decay of HFEs. This would lead to an inflation of the impact of HFEs on the fixation probability of the introgressed B allele in small populations. To counter this effect and allow an assessment of the stochastic dilution of introgressed DNA, we only allow the inheritance of introgressed DNA alongside the introgressed B allele.

Fixation probabilities from a branching process

Here, we will derive an expression for fixation probability under consideration of HFEs using a branching process (Taylor and Karlin 1998). We suppose an infinitely large, randomly mating population of diploid individuals and that the tracked B allele is initially present as a single copy, i.e., the hybridization of a single individual from the donor and recipient population. Furthermore, we assume that each parent allele independently contributes k offspring alleles to the next generation, where k is Poisson distributed. Then, the probability generating function for the number of offspring alleles in generation t is:

$$Pr_t(z) = e^{\lambda_t (z-1)} \quad \text{(S4)}$$

where λ_t is the hybrid fitness $w_{AB,t}$ in generation t. Due to the time-varying hybrid fitness, compounding the probability generating function is time-heterogeneous, and the probability generating function of the number of offspring alleles after t generations is

$$G_t(z) = exp\left[\lambda_0 \left(...\lambda_{t-3} \left(exp\left(\lambda_{t-2} \left(exp\left[\lambda_{t-1}(z-1)\right] - 1\right)\right) - 1\right)...\right)\right] \quad \text{(S5)}$$

Because the only two absorbing states are extinction and fixation of an allele, they are implicit of each other, and the fixation probability of an allele is equivalent to its survival probability (u). The probability of extinction by generation t is $G_t(0)$, and thus the survival probability, that is, there is at least one copy in generation t, is

$$u_t = 1 - G_t(0) = 1 - exp\left[\lambda_0 \left(...\lambda_{t-3} \left(exp\left(\lambda_{t-2} \left(exp\left[-\lambda_{t-1}\right] - 1\right)\right) - 1\right)...\right)\right] \quad \text{(S6)}$$

This expression was derived by Ohta and Kojima (1968) for the survival probability of an inversion with time-varying fitness. Due to the time-varying fitness, the fixation probability must usually be approximated numerically from Equation S6 (Ohta and Kojima 1968; Uecker and Hermisson 2011). Equation S6 is the same as Equation 8 in the main text.

Different epistasis models leading to DMI effects

In the main text, we focused on DMI effects arising from epistatic interactions between unlinked semidominant introgressed and semidominant non-introgressed alleles (Equation 5). As pointed out in the main text, while the dynamics are identical if introgressed alleles are dominant, no DMI effects are observed in the scenario of a rare introgression event if introgressed alleles are recessive. However, the dynamics differ if non-introgressed alleles are recessive or dominant. Generally, the overall conclusions drawn in the main text regarding the impact of HFEs on the fate of introgressed alleles remain unchanged for the different models of epistatic interactions, but the dynamics slightly differ. Here, we will discuss the dynamics under these different models of epistatic interactions.

To distinguish the dynamics under the different epistasis models, we refer to the model discussed in the main text as the semidominance-dominance epistasis model (δ_t), to the model of interactions between recessive non-introgressed alleles and (semi-)dominant introgressed alleles as the recessive-dominance epistasis model ($\delta_{rd,t}$), and to the model of interactions between dominant non-introgressed alleles and (semi-)dominant introgressed alleles as the dominance-dominance epistasis model ($\delta_{dd,t}$). These three epistasis models differ in the decay rates and onset of DMI effects.

The logic of the decay of DMI effects under the recessive-dominance and dominance-dominance epistasis model is the same as for the semidominance-dominance epistasis model, which is described in detail above. However, their decay functions (g_t) are different. As a reminder, we presume the introgression of a single set of chromosomes with n loci, and the amount of introgressed DNA is assumed to halve every generation. We also presume polygenic DMI effects as per empirical results of True *et al.* (1996) and Presgraves and Meiklejohn (2021). Hence, each DMI makes a small contribution to the overall DMI effects, and the strength of DMI effects decays with the dilution of introgressed DNA in hybrid genomes. Above, we showed that in this case, the multiplicative model of DMI effects is approximated by the first term of its Taylor series, which is equivalent to an additive model.

Under the recessive-dominance epistasis model, interactions between introgressed and non-introgressed loci do not occur in the F1 hybrid (Figure S4). This is because the F1 hybrid cannot be homozygous for non-introgressed loci. Due to repeated backcrossing within the recipient population, hybrids become homozygous for non-introgressed loci so that DMI effects become possible. For this reason, DMI effects are observed first in BC1 hybrids ($\delta_{rd,2}$). In BC1 hybrids, $\frac{1}{2}n$ introgressed loci can interact with the $\frac{1}{2}n$ non-introgressed loci for which BC1 hybrids are homozygous, amounting to $\frac{1}{2}n \times \frac{1}{2}n = \frac{1}{4}n^2$ possible interactions. Extending this logic to BC2 and BC3 hybrids, $\frac{3}{4}n \times \frac{1}{4}n = \frac{3}{16}n^2$ and $\frac{7}{8}n \times \frac{1}{8}n = \frac{7}{64}n^2$ interactions are possible. Therefore, the decay of DMI effects in the scenario of the recessive-dominance epistasis model is given by:

$$\delta_{rd,t} = \delta_{rd,2} \times \left[(2^t - 2)2^{3-2t}\right] \tag{S7}$$

Thus the decay rate under the recessive-dominance epistasis model is the same as under the semidominance-dominance epistasis model, but DMI effects are delayed by one generation under the recessive-dominance (compare Figure 1B & Figure S4). When the initial strengths of DMI effects under the semidominance-dominance and recessive-dominance epistasis models are the same ($\delta_1 = \delta_{rd,2}$), the compounded DMI effects are identical.

Under the dominance-dominance epistasis model, the number of possible interactions between introgressed and non-introgressed alleles inherited from different parents determines the strength of DMI effects. Interactions between introgressed and non-introgressed alleles inherited from the same parent do not add to DMI effects because of the dominance of non-introgressed loci (Figure S5). Thus, the number of possible interactions simply halves every generation (i.e., n^2 in F1 hybrids, $\frac{1}{2}n^2$ in BC1 hybrids, etc.). Hence, the first part in the parenthesis of the non-reduced form of Equation 5 describes the decay of DMI effects under this model ($\delta_{dd,t}$):

$$\delta_{dd,t} = \delta_{dd,1} \times 2^{-(t-1)} \tag{S8}$$

where $\delta_{dd,1}$ is the strength of DMI effects in the F1 hybrid. Note that the decay rate of DMI effects under the dominance-dominance epistasis model and the decay of heterosis effects is the same.

Table S2 summarizes the decay of the expected strength of DMI effects for each epistasis model up to BC4 hybrids. m is the expected number of DMIs in the F1/BC1 hybrid (i.e., the number of possible interactions in the F1/BC1 hybrid times the probability that an interaction is incompatible), and c is the effect size of an individual DMI.

Hybrid fitnesses are similarly influenced by the different epistasis models. When explaining DMI effects with the recessive-dominance epistasis model, DMI effects show the same decay rate but are delayed by one generation compared to under the semidominance-dominance epistasis model (Figure S6). Because of this, the hybrid breakdown in BC1 hybrids is more severe under the recessive-dominance epistasis model compared to the semidominance-dominance epistasis model, given the same initial strength of DMI effects ($\delta_1 = \delta_{rd,2}$). In contrast to this, the dynamics are simpler when assuming the dominance-dominance epistasis model to explain DMI effects (Figure S7). Due to the same decay rates of heterosis and DMI effects, either hybrid vigor (i.e., when $\eta_1 > |\delta_{dd,1}|$) or hybrid breakdown (i.e., when $|\delta_{dd,1}| > \eta_1$) is observed, but no hybrid vigor followed by hybrid breakdown can be observed. Heterosis and DMI effects cancel out under the dominance-dominance epistasis model when heterosis and DMI effects have the same initial strength ($\eta_1 = |\delta_{dd,1}|$).

Fixation probabilities are similarly affected by the different epistasis models. The accuracy of Equations 7 & 8, respectively, is robust to the choice of epistasis model (compare Figures 3, S8 & S9). Under the recessive-dominance epistasis model, fixation probabilities are increased when initial heterosis effects are more than 1.62 times as strong as DMI effects ($\eta_1 > 1.62 \times |\delta_{rd,2}|$; Figure S10). This deviates from the expected condition for weak HFEs, i.e., the fixation probability is increased if heterosis effects are initially $\frac{4}{3}$ times as strong as DMI effects (see Results in the main text). This is because initial heterosis effects need to be stronger to overcome DMI effects than under the semidominance-dominance epistasis model since hybrid breakdown in the BC1 and following generations is more severe due to the DMI effects being delayed by one generation. In contrast to this, under the dominance-dominance epistasis model, fixation probabilities are always increased when initial heterosis effects are stronger than DMI effects ($\eta_1 > |\delta_{dd,1}|$) and vice versa. This finding is due to identical decay rates under this epistasis model (Figure S11).

The distribution of fitness effects (DFE) of alleles that reach fixation is similarly transformed by the different epistasis models for DMI effects. Under the recessive-dominance epistasis model, the DFE is shifted to slightly greater selection coefficients when DMI effects are strong ($\eta_1 = 0.0$; $\delta_{rd,1} = -0.8$), i.e., $\bar{s}$ is 0.0649 compared to 0.0634 under the semidominance-dominance epistasis model (Figure S12). In contrast to this, under the dominance-dominance epistasis model, the shift towards greater selection coefficients is diminished when DMI effects are strong ($\eta_1 = 0.0$; $\delta_{dd,1} = -0.8$), i.e., $\bar{s}$ is 0.0633 compared to 0.0634 under the semidominance-dominance epistasis model (Figure S13).

Sojourn times are also qualitatively similarly affected by the different epistasis models for DMI effects. Under the recessive-dominance

epistasis model, the loss of an allele is accelerated due to stronger hybrid breakdown compared to the semidominance-dominance epistasis model (compare Figure 6D & S14D). Contrarily, under the dominant-dominant epistasis model, sojourn times under the classical model are matched if heterosis and DMI effects have the same strength ($\eta_1 = |\delta_{dd,1}|$, Figure S15).

Literature cited

Ohta T, Kojima KI. 1968. Survival Probabilities of New Inversions in Large Populations. Biometrics. 24:501.

Presgraves DC, Meiklejohn CD. 2021. Hybrid Sterility, Genetic Conflict and Complex Speciation: Lessons From the Drosophila simulans Clade Species. Frontiers in Genetics. 12:1–18.

Taylor H, Karlin S. 1998. *An Introduction to Stochastic Modeling*. Academic Press.

True JR, Weirt BS, Laurie CC. 1996. A Genome-Wide Survey of Hybrid Incompatibility Factors by the Introgression of Marked Segments of Drosophila mauritiana Chromosomes into Drosophila simulans. Technical report.

Uecker H, Hermisson J. 2011. On the Fixation Process of a Beneficial Mutation in a Variable Environment. Genetics. 188:915.

Table S1 Comparison of fixation probability if the amount of introgressed donor DNA - and hence also HFEs - decays deterministically (i.e., halves every generation) or stochastically (i.e., Equation S3). Fixation probabilities were computed based on $1,000,000$ WF simulations with $s = 0.01$. Statistically significant different fixation probabilities are indicated by * (Fisher's exact test, $P < \frac{0.05}{8} = 0.00625$).

	Strong heterosis ($\eta_1 = 0.8,\ \delta_1 = 0.0,\ \alpha = 3.67$)		Strong DMI effects ($\eta_1 = 0.0,\ \delta_1 = -0.8,\ \alpha = 0.035$)	
Population size / dilution model	Stochastic	Deterministic	Stochastic	Deterministic
$N_e = 100$	0.067747	0.067845	0.000830	0.000730
$N_e = 500$	0.070015*	0.068830*	0.000923*	0.000761*
$N_e = 1000$	0.067906*	0.069135*	0.000765	0.000718
$N_e = 10000$	0.068043	0.068843	0.000767	0.000670

Table S2 Decay of the expected strength of DMI effects under different epistasis models

Hybrid generation / epistasis model	F1	BC1	BC2	BC3	BC4
Semidominance-dominance (δ_t)	$-mc$	$-\frac{3}{4}mc$	$-\frac{7}{16}mc$	$-\frac{15}{64}mc$	$-\frac{31}{256}mc$
Recessive-dominance ($\delta_{rd,t}$)	0	$-mc$	$-\frac{3}{4}mc$	$-\frac{7}{16}mc$	$-\frac{15}{64}mc$
Dominance-dominance ($\delta_{dd,t}$)	$-mc$	$-\frac{1}{2}mc$	$-\frac{1}{4}mc$	$-\frac{1}{8}mc$	$-\frac{1}{16}mc$

Supplementary Figures

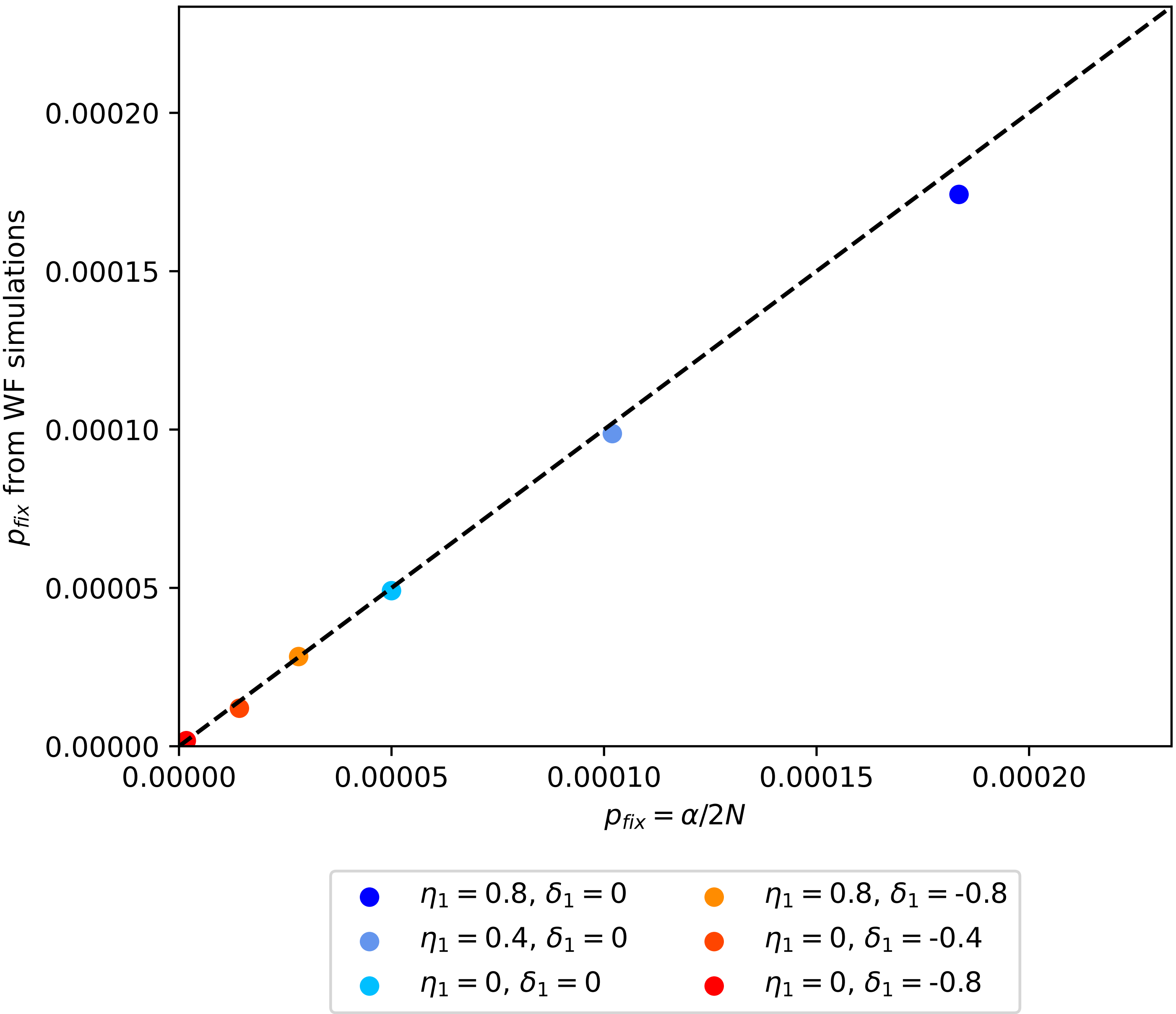

Figure S1 Comparison of the fixation probabilities of a neutral introgressed B approximated by $\alpha/2N$ and derived from WF simulations for different scenarios. The root mean square error is less than 10^{-10}. The fixation probabilities inferred from WF simulations were estimated based on 10^8 runs assuming an effective population size of $N_e = 10,000$ and an initial allele frequency of $q_0 = 1/2N_e$.

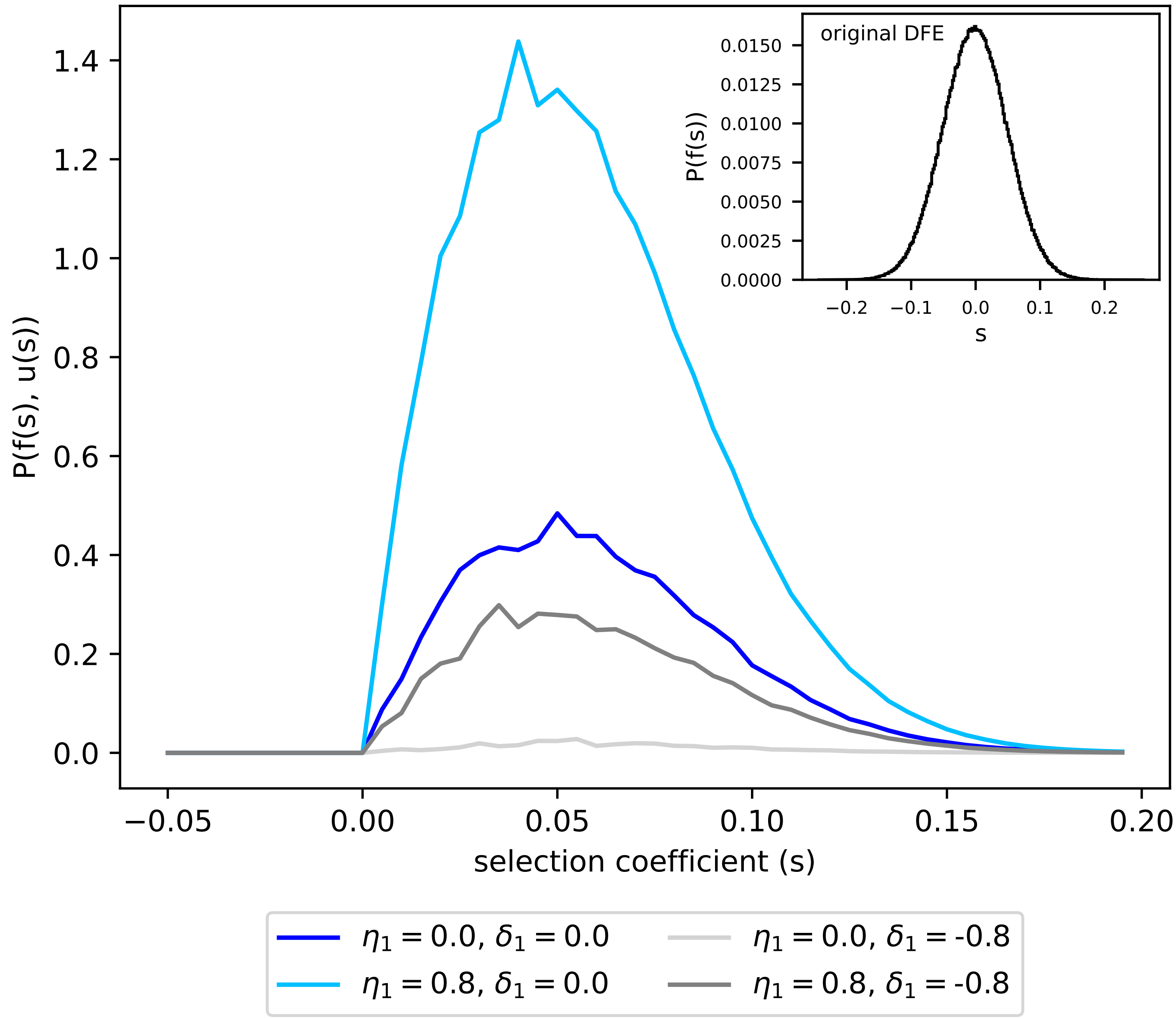

Figure S2 The joint distribution of fitness effects and fixation probabilities, i.e., $2\alpha s f(s)$. HFEs (as encompassed by the parameter α) linearly transform the joint distribution independently from the original shape of the DFE. As represented by the area under the curve, heterosis effects increase the overall likelihood of introgression, while DMI effects diminish it. The original DFE of alleles was modeled with a normal distribution with a mean of -0.001 and a standard deviation of 0.05. Fixation probabilities of the introgressed B allele were estimated based on 100,000 Wright-Fisher simulations assuming an effective population size of $N_e = 10,000$ and an initial allele frequency of $q_0 = 1/2N_e$.

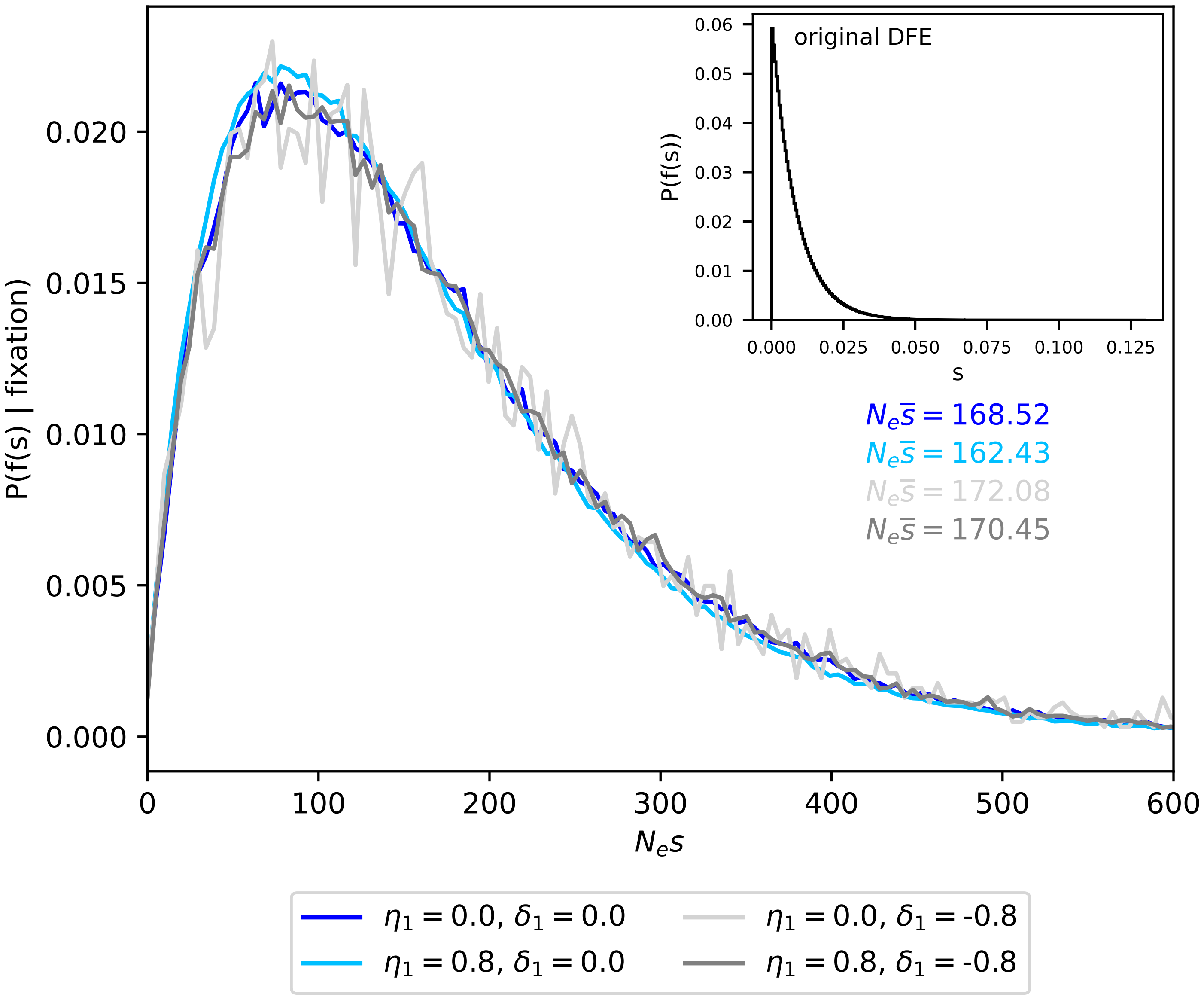

Figure S3 The distribution of fitness effects of introgressed alleles conditioned on fixation when the original DFE follows an exponential distribution (see inset). The mean of the original DFE was set to 0.0085. The observed trends are the same as when modeling the original DFE with a normal distribution (compare Figure 5). Heterosis effects subtly shift the DFE towards smaller selection coefficients, while DMI effects shift it to slightly greater values. Under the classical model, $N_e\bar{s}$ is 168.52. DMI effects increase it to 172.08, while heterosis effects reduce it to 162.43. When heterosis and DMI effects are of similar initial absolute strength, $N_e\bar{s}$ increases to 170.45. The DFEs of fixed alleles was inferred from 10^7 Wright-Fisher simulations with selection coefficients drawn from the original DFE. Simulation parameters: $N_e = 10,000$ and $q_0 = 1/2N_e$.

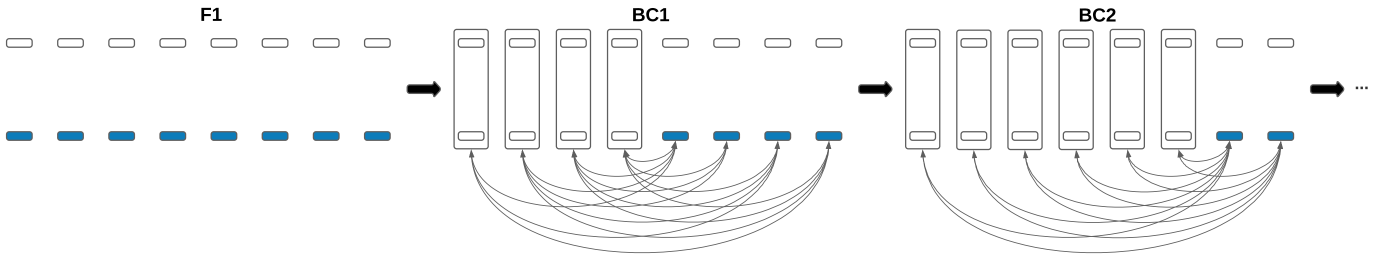

Figure S4 Illustration of the decay of possible effects between unlinked dominant introgressed (blue) and recessive non-introgressed (white) alleles causing DMI effects. Because of the recessive non-introgressed alleles interactions between introgressed and non-introgressed loci are not occurring in the F1 hybrid. Interactions are first observed in BC1 hybrids. The subsequent decay of DMIs is the same as under the semidominance-dominance epistasis model. The number of possible incompatible interactions does not halve every generation, as the alleles for which the hybrids become homozygous interact in with other unlinked alleles, too. (see Equation S7 in the main text). The boxes indicate homozygosity for recessive non-introgressed loci.

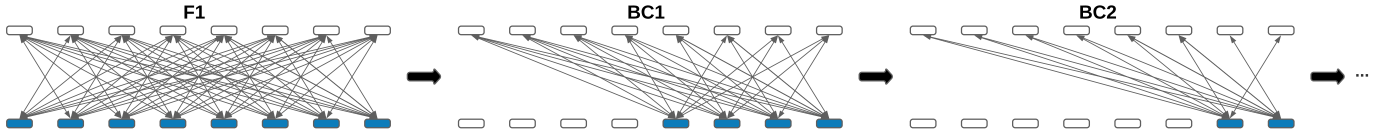

Figure S5 Illustration of the decay of possible effects between unlinked dominant introgressed (blue) and dominant non-introgressed (white) alleles causing DMIs. The number of possible incompatible interactions halves every generation (see Equation S8 in the main text). Due to the complete dominance of the non-introgressed loci, interactions between introgressed and non-introgressed loci inherited from the same parent can be neglected.

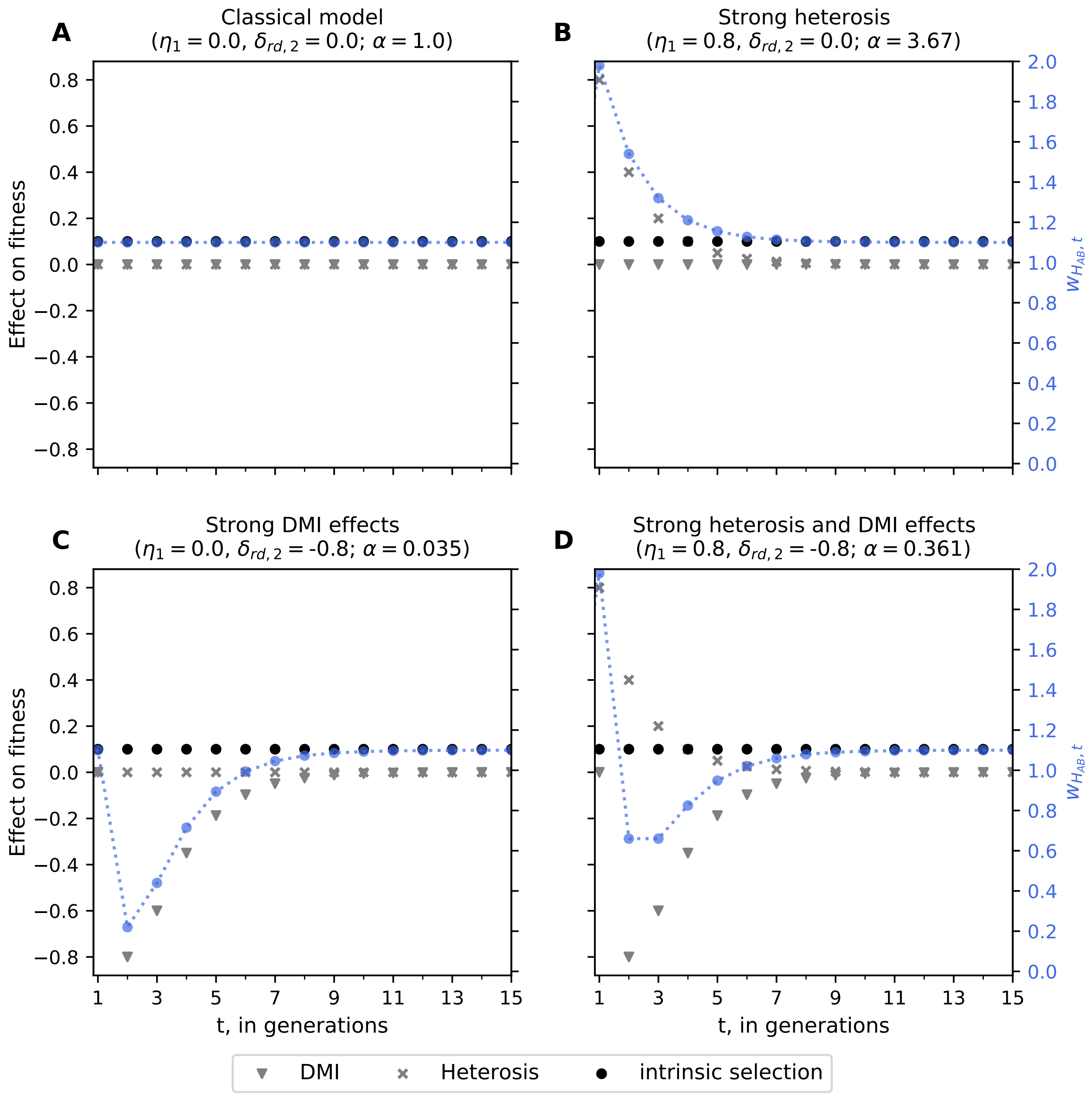

Figure S6 Temporal dynamics of different hybrid fitness scenarios where DMI effects arise from recessive-dominance epistasis: **A**) classical model, **B**) strong heterosis effects, **C**) strong DMI effects, and **D**) strong heterosis and DMI effects. HFEs (greyscale) quickly decay and asymptotically approach zero. Therefore, the overall fitness of heterozygotes that carry the introgressed *B* allele (blue) is time-sensitive. In the absence of HFEs, our model is equivalent to the classical model with constant hybrid fitness determined by the intrinsic selection of the tracked *B* allele ($s = 0.1$; black). When $\eta_1 > |\delta_{rd,2}|$, heterosis is initially the dominant force, resulting in hybrid vigor until intrinsic selection takes over after approximately eight generations. Conversely, when $|\delta_{rd,2}| > \eta_1$, DMI effects are the stronger force, leading to hybrid breakdown, until intrinsic selection takes over. When $\eta_1 = |\delta_{rd,2}|$, heterosis effects are the stronger evolutionary force in the F1 hybrid, inducing hybrid vigor. With the onset of DMI effects in BC1 hybrids, it comes to severe hybrid breakdown. α is the compounded effect size of HFEs (Eq. 6).

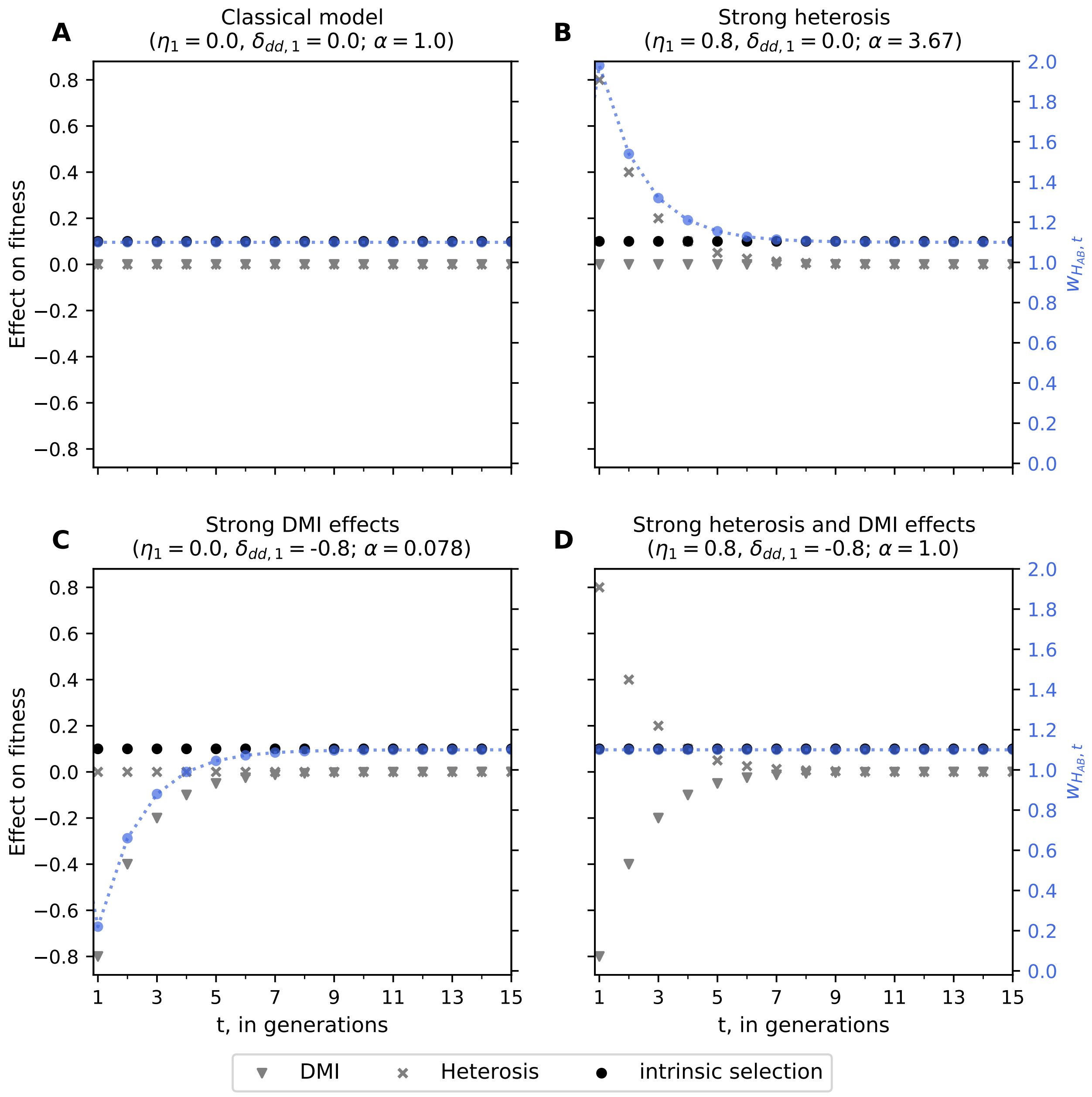

Figure S7 Temporal dynamics of different hybrid fitness scenarios where DMI effects arise from dominance-dominance epistasis: **A**) classical model, **B**) strong heterosis effects, **C**) strong DMI effects, and **D**) strong heterosis and DMI effects. HFEs (greyscale) quickly decay and asymptotically approach zero. Therefore, the overall fitness of heterozygotes that carry the introgressed B allele (blue) is time-sensitive. In the absence of HFEs, our model is equivalent to the classical model with constant hybrid fitness determined by the intrinsic selection of the tracked B allele ($s = 0.1$; black). When $\eta_1 > |\delta_{dd,1}|$, heterosis is initially the stronger force, resulting in hybrid vigor until intrinsic selection takes over after approximately eight generations. Conversely, when $|\delta_{dd,1}| > \eta_1$, DMI effects are the stronger force, leading to hybrid breakdown, until intrinsic selection takes over. When $\eta_1 = |\delta_{dd,1}|$, heterosis effects and DMI effects cancel each other because of the same decay rates. Thus, our model resembles the classical model. α is the compounded effect size of HFEs (Eq. 6).

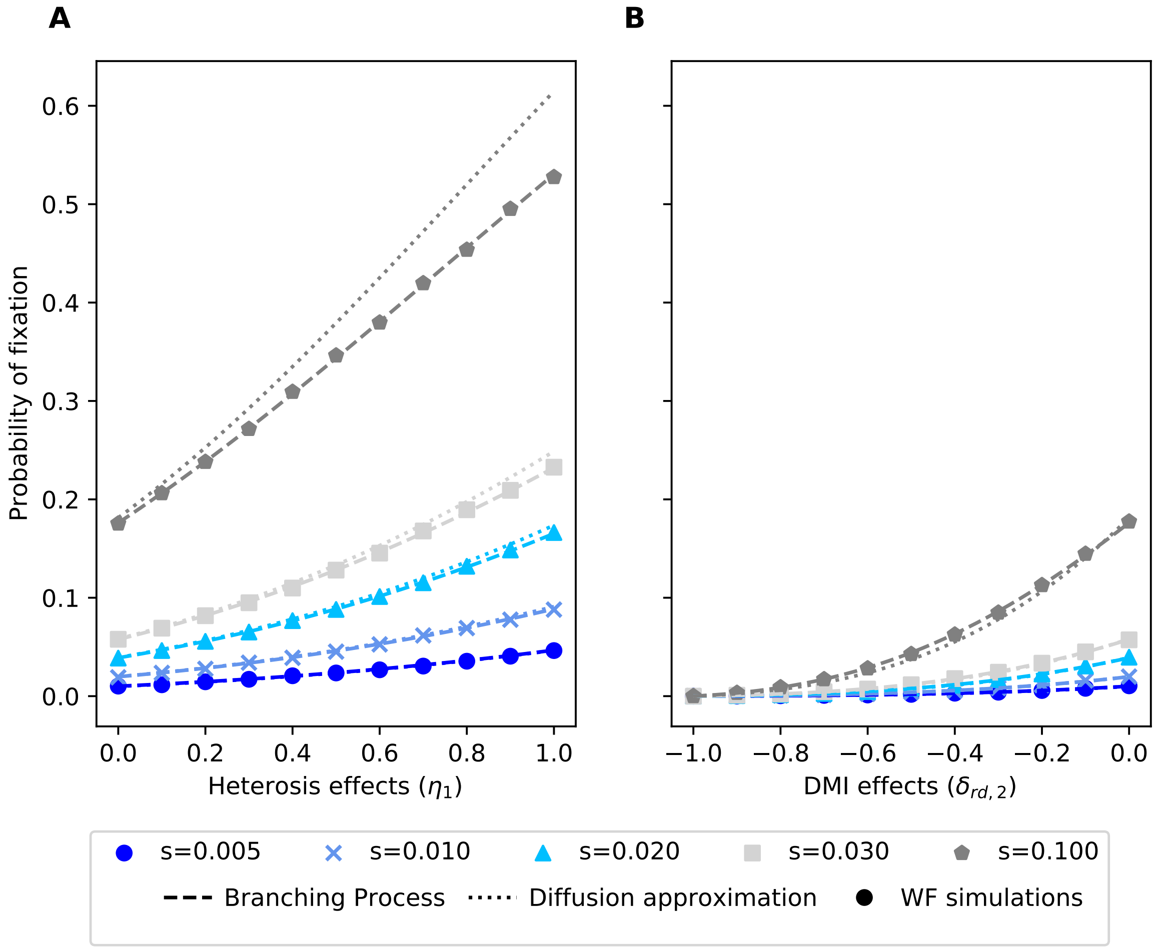

Figure S8 Comparison of the fixation probabilities of the introgressed B allele obtained from diffusion approximations (Eq. 7), branching processes (Eq. 8), and Wright-Fisher simulations when DMI effects arise from recessive-dominance epistasis. **A)** only heterosis effects ($\delta_{rd,2} = 0.0$) and **B)** only DMI effects ($\eta_1 = 0.0$). Theoretical fixation probabilities match simulations, with the exception of the diffusion approximation of the fixation probability for strongly selected alleles in the presence of strong heterosis. Fixation probabilities were computed based on $100,000$ simulations with $N_e = 10,000$ and $q_0 = 1/2N_e$.

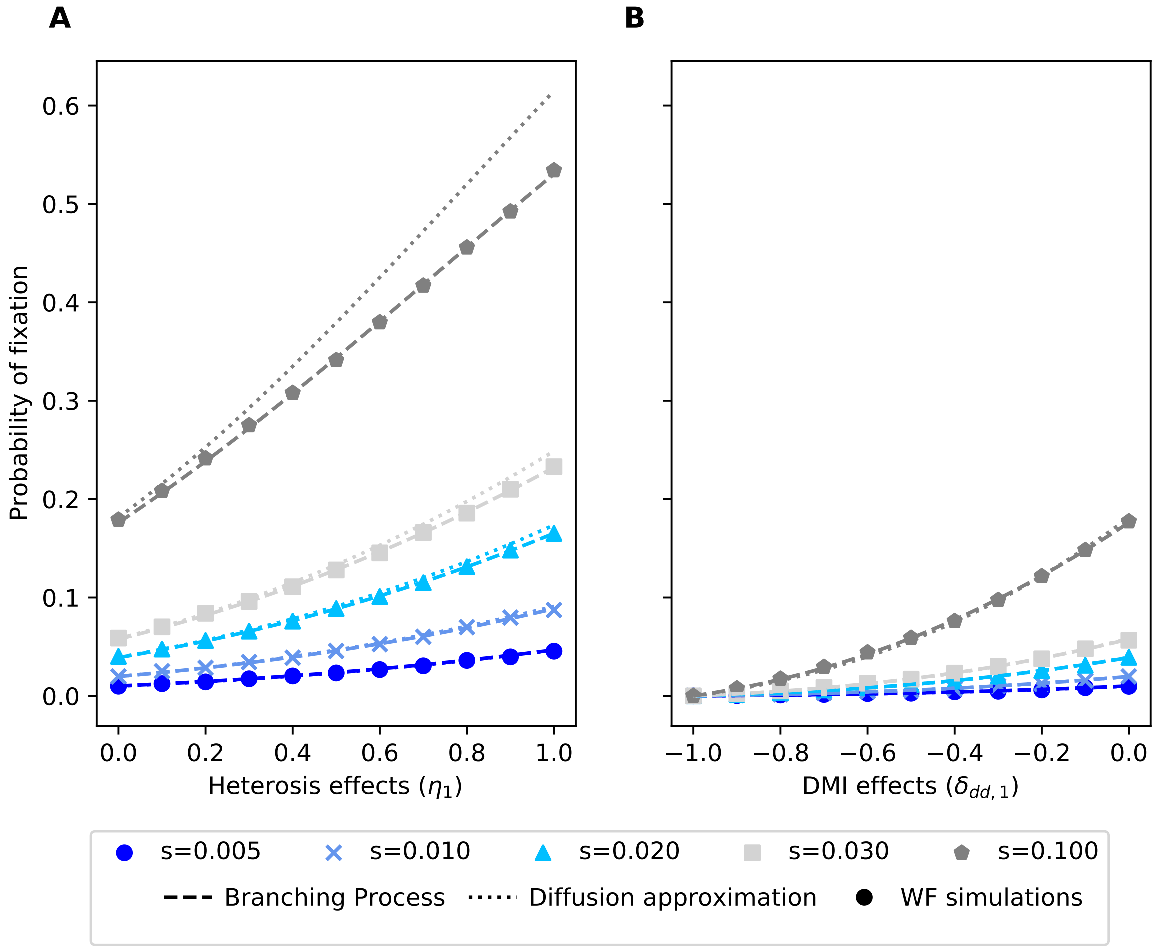

Figure S9 Comparison of the fixation probabilities of the introgressed B allele obtained from diffusion approximations (Eq. 7), branching processes (Eq. 8), and Wright-Fisher simulations when DMI effects arise from dominance-dominance epistasis.. **A)** only heterosis effects ($\delta_{dd,1} = 0.0$) and **B)** only DMI effects ($\eta_1 = 0.0$). Theoretical fixation probabilities match simulations, with the exception of the diffusion approximation of the fixation probability for strongly selected alleles in the presence of strong heterosis. Fixation probabilities were computed based on 100,000 simulations with $N_e = 10,000$ and $q_0 = 1/2N_e$.

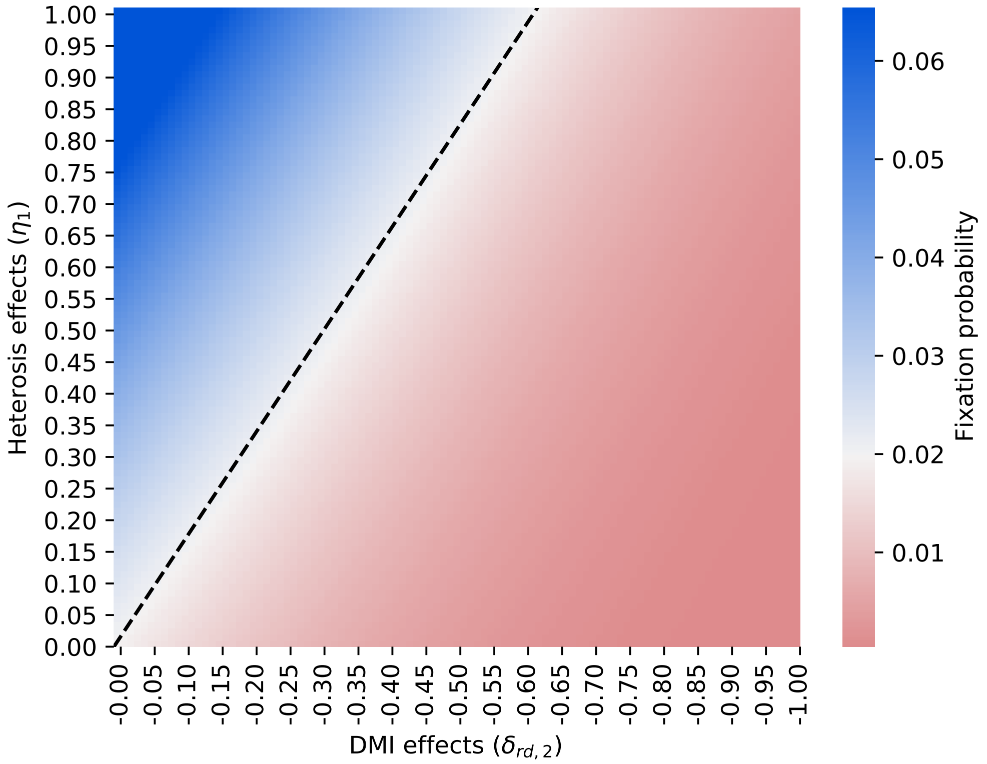

Figure S10 Fixation probabilities for different initial strengths of heterosis effects and recessive-dominant DMI effects. Heterosis effects can increase the fixation probability up to $\sim$ 4-fold (top-left corner), while DMI effects can prohibit fixation (bottom-right corner). The black line corresponds to parameter combinations that match fixation probabilities expected from standard theory ($\sim 2s$). Due to different decay rates of heterosis effects and DMI effects under the recessive-dominance epistasis model, this line does not follow the diagonal. The slope of the line is $\sim$ 1.62, and hence steeper than the slope of the line under the semidominance-dominance epistasis model (1.35, compare Figure 4 in the main text), representing the more severe hybrid breakdown under this epistasis model for DMI effects. Thus, fixation probabilities are increased by HFEs when $\eta_1 > 1.62 \times |\delta_{rd,2}|$ ($R^2 = 0.99,\ P < 10^{-10}$). Fixation probabilities for the B allele with a selection coefficient $s = 0.01$ were computed using Equation 8.

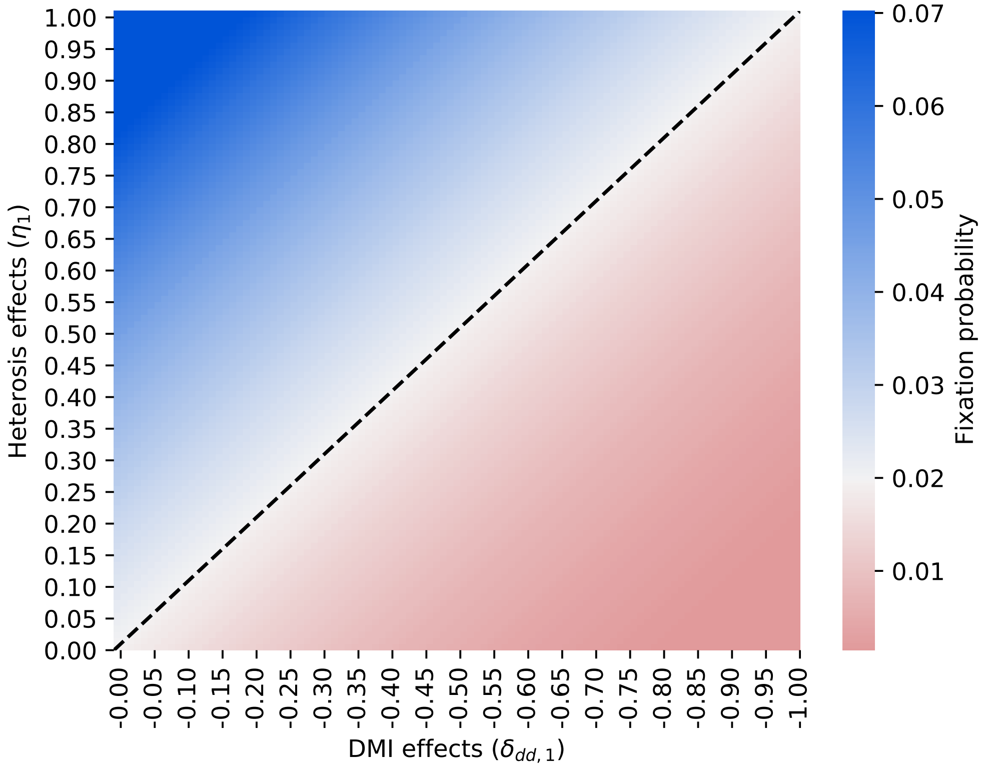

Figure S11 Fixation probabilities for different initial strengths of heterosis effects and dominant-dominant DMI effects. Heterosis effects can increase the fixation probability up to $\sim$ 4-fold (top-left corner), while DMI effects can prohibit fixation (bottom-right corner). The black line corresponds to parameter combinations that match fixation probabilities expected from standard theory ($\sim 2s$). Because of the same decay rates of heterosis effects and DMI effects under the dominance-dominance epistasis model, this line does follow the diagonal. The classical model is resembled when $\eta_1 = |\delta_{dd,1}|$. Fixation probabilities are increased when $\eta_1 > |\delta_{dd,1}|$, whereas it is decreased when $|\delta_{dd,1}| > \eta_1$ ($R^2 = 1.00; P < 10^{-10}$). Fixation probabilities for the B allele with a selection coefficient $s = 0.01$ were computed using Equation 8.

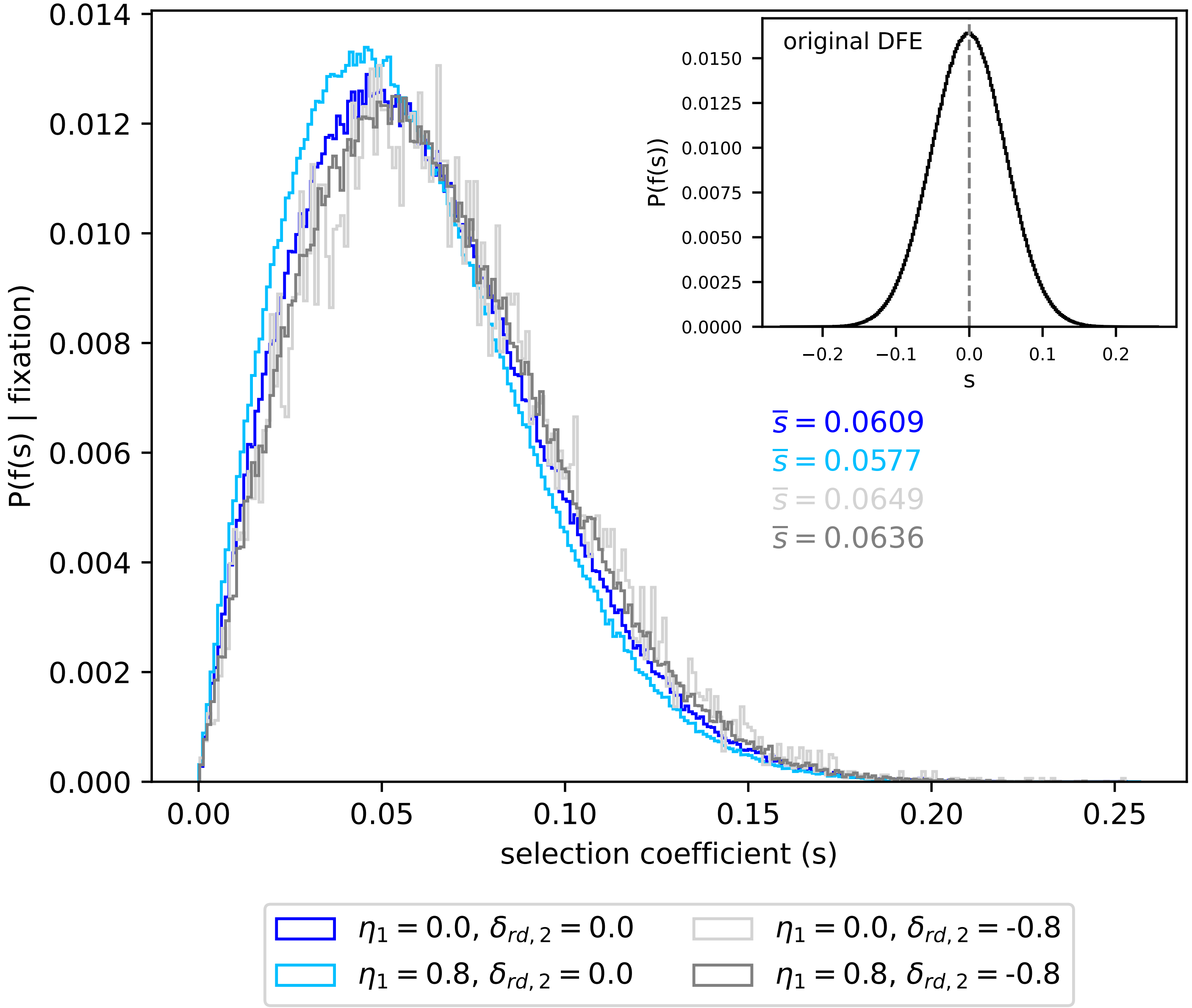

Figure S12 The distribution of fitness effects of introgressed alleles conditioned on fixation when the original DFE is modeled with a normal distribution with mean -0.001 and standard deviation 0.05, and DMI effects arise from recessive-dominance epistasis. Heterosis effects subtly shift the DFE towards smaller selection coefficients, while DMI effects shift it towards slightly greater values. Under this model, the shift is slightly more prominent than under the semidominance-dominance epistasis model due to the stronger hybrid breakdown (compare Figure 5). The mean selection coefficient is shifted from 0.0609 under the classical model to 0.0636 (compared to 0.0622 if semidominance-dominance epistasis is assumed) when $\eta_1 = 0.8$ and $\delta_{rd,2} = \delta_{sd,1} = -0.8$. The DFEs of fixed alleles was inferred from 10^7 Wright-Fisher simulations with selection coefficients drawn from the original DFE (see inset). Simulation parameters: $N_e = 10,000$ and $q_0 = 1/2N_e$.

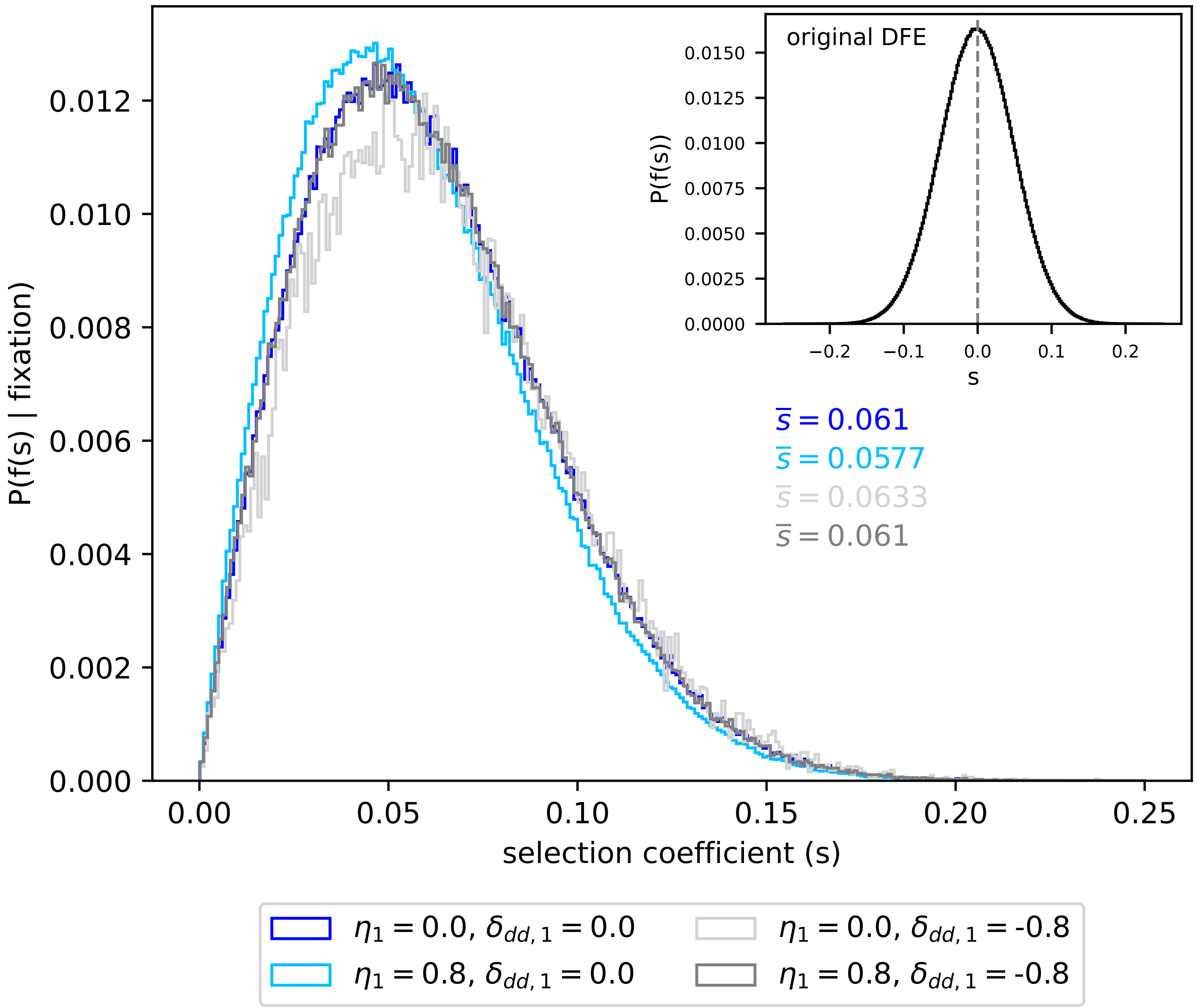

Figure S13 The distribution of fitness effects of introgressed alleles conditioned on fixation when the original DFE is modeled with a normal distribution with mean -0.001 and standard deviation 0.05, and DMI effects arise from dominance-dominance epistasis. Heterosis effects subtly shift the DFE towards smaller selection coefficients, while DMI effects shift it towards slightly greater values. Under this model, the shift is slightly less prominent than under the semidominance-dominance epistasis model due to the weaker hybrid breakdown (compare Figure 5). The mean selection coefficient is shifted from 0.0610 under the classical model to 0.0633 (compared to 0.0634 if semidominance-dominance epistasis is assumed) when $\eta_1 = 0.0$ and $\delta_{dd,1} = \delta_{sd,1} = -0.8$. Note that the curves for the cases when $\eta_1 = \delta_{dd,1} = 0.0$ and when $\eta_1 = |\delta_{dd,1}| = 0.8$ overlap because both resemble the classical model. The DFEs of fixed alleles was inferred from 10^7 Wright-Fisher simulations with selection coefficients drawn from the original DFE (see inset). Simulation parameters: $N_e = 10,000$ and $q_0 = 1/2N_e$.

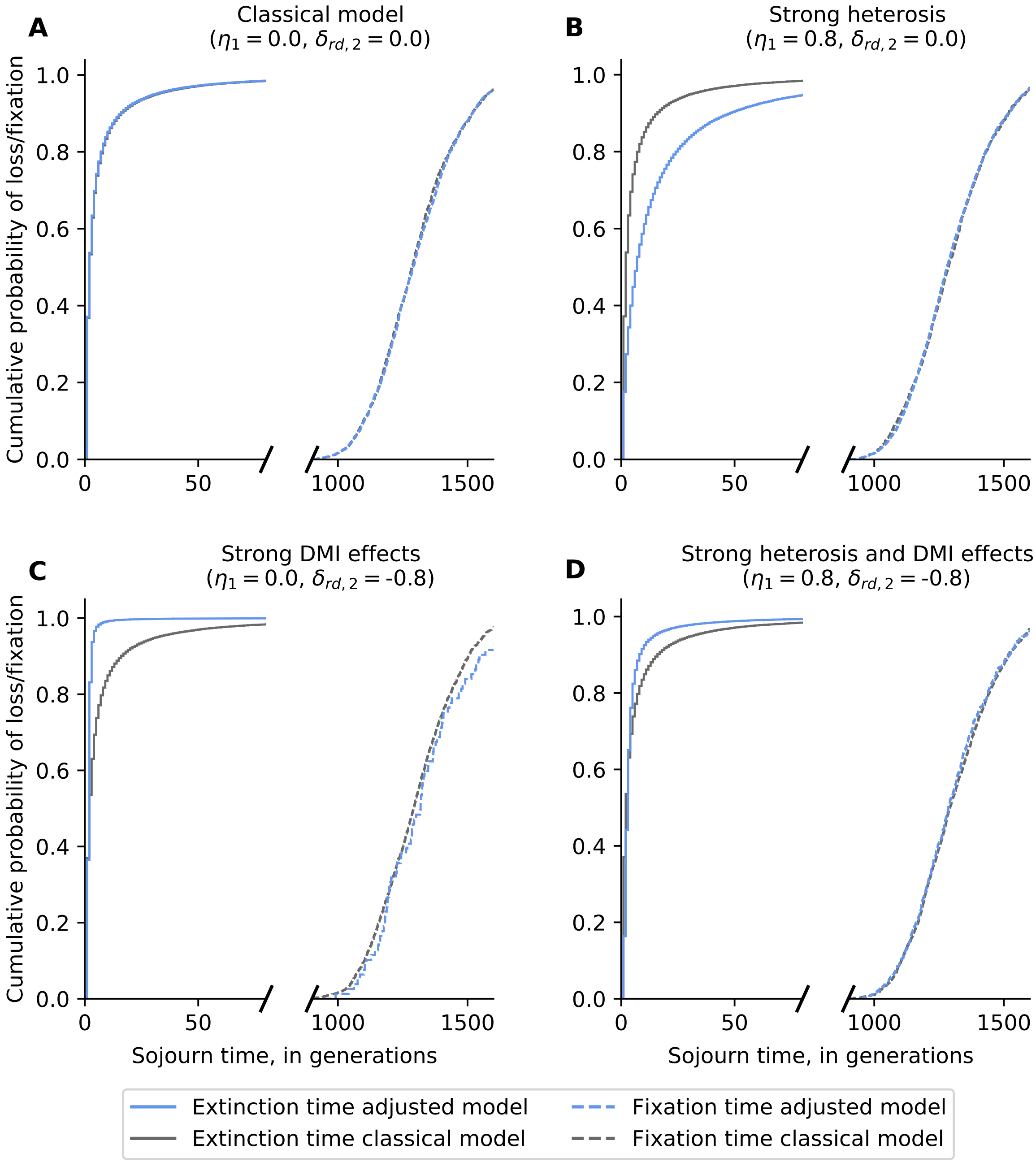

Figure S14 HFEs affect extinction times but not fixation times when DMI effects arise from recessive-dominance epistasis: **A**) classical model, **B**) strong heterosis effects, **C**) strong DMI effects, and **D**) strong heterosis and DMI effects. Strong heterosis effects prolong the time until the loss of the introgressed B allele, whereas strong DMI effects expedite its loss compared to the classical model. When heterosis effects and DMI effects are of similar strength, the extinction is slightly expedited due to the slower decay of DMI effects. This is because HFEs act on a shorter timescale than on which an allele reaches fixation, illustrating the separation of timescales. The differences between our model and the classical model regarding the extinction time are slightly more distinct under this epistasis model than compared to the semidominance-dominance epistasis model (compare Figure 6D in the main text). Distributions of sojourn times were inferred based on 100,000 Wright-Fisher simulations with $N_e = 10,000$, $s = 0.01$, and $q_0 = 1/2N_e$.

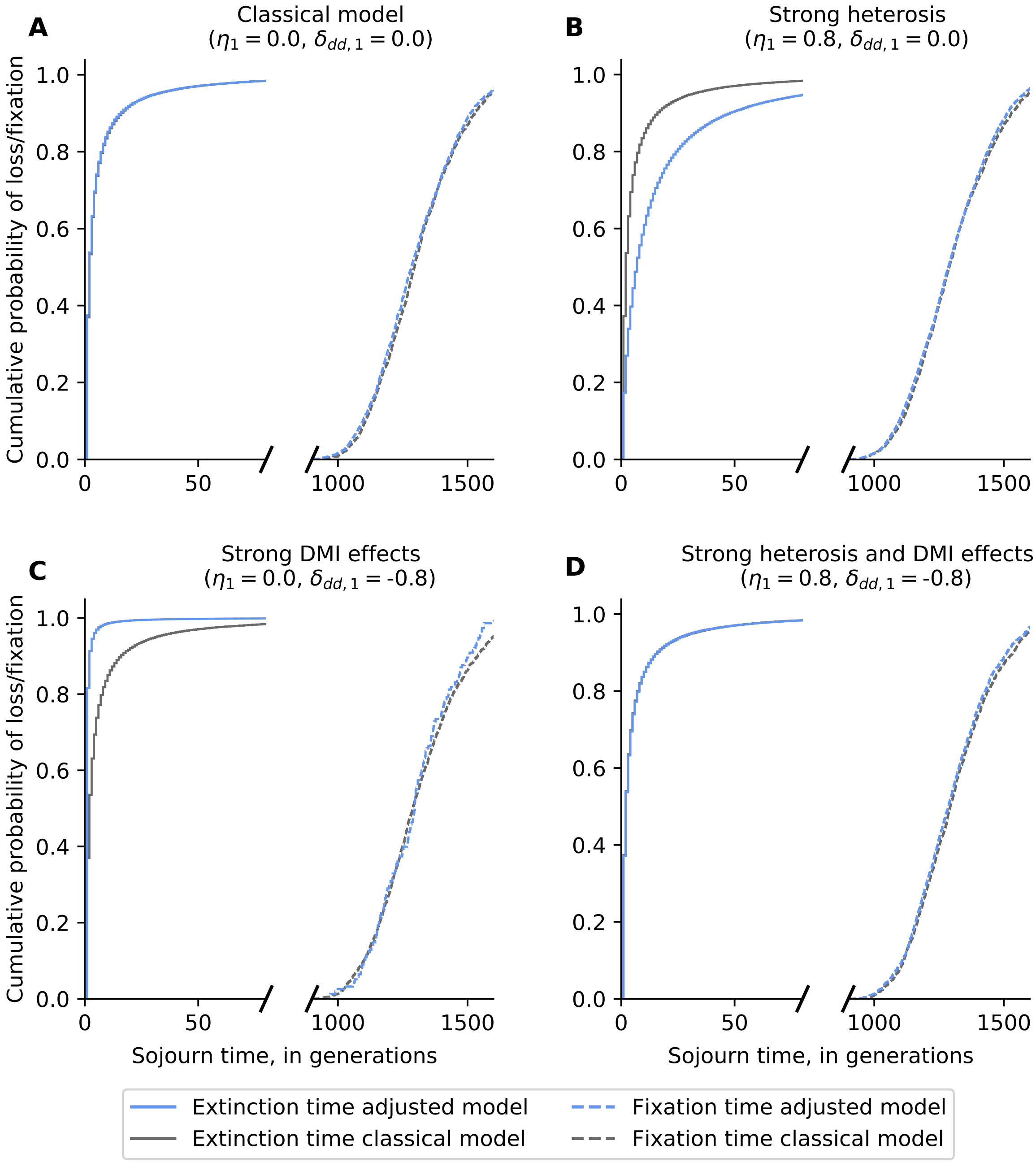

Figure S15 HFEs affect extinction times but not fixation times when DMI effects arise from dominance-dominance epistasis: **A**) classical model, **B**) strong heterosis effects, **C**) strong DMI effects, and **D**) strong heterosis and DMI effects. Strong heterosis effects prolong the time until the loss of the introgressed *B* allele, whereas strong DMI effects expedite its loss compared to the classical model. This is because HFEs act on a shorter timescale than on which an allele reaches fixation, illustrating the separation of timescales. When heterosis effects and DMI effects are of the same strength, heterosis and DMI effects cancel each other out so that our model resembles the classical model. Distributions of sojourn times were inferred based on $100,000$ Wright-Fisher simulations with $N_e = 10,000$, $s = 0.01$, and $q_0 = 1/2N_e$.
